# 2-Butoxytetrahydrofuran, Isolated from *Holothuria scabra*, Attenuates Aggregative and Oxidative Properties of α-Synuclein and Alleviates Its Toxicity in a Transgenic *Caenorhabditis elegans* Model of Parkinson’s Disease

**DOI:** 10.1021/acschemneuro.4c00008

**Published:** 2024-05-10

**Authors:** Sukrit Promtang, Tanatcha Sanguanphun, Pawanrat Chalorak, Laurence S. Pe, Nakorn Niamnont, Prasert Sobhon, Krai Meemon

**Affiliations:** † Molecular Medicine Program, Multidisciplinary Unit, Faculty of Science, Mahidol University, Ratchathewi, Bangkok 10400, Thailand; ‡ Department of Anatomy, Faculty of Science, Mahidol University, Ratchathewi, Bangkok 10400, Thailand; § Department of Radiological Technology and Medical Physics, Faculty of Allied Health Sciences, Chulalongkorn University, Pathumwan, Bangkok 10330, Thailand; ∥ Research Center for Neuroscience, Institute of Molecular Biosciences, Mahidol University, Salaya, Nakhon Pathom 73170, Thailand; ⊥ Department of Chemistry, Faculty of Science, King Mongkut’s University of Technology Thonburi, Bang Mod, Bangkok 10140, Thailand; # Center for Neuroscience, Faculty of Science, Mahidol University, Ratchathewi, Bangkok 10400, Thailand

**Keywords:** 2-butoxytetrahydrofuran, *Holothuria scabra*, antiaggregation, antioxidation, α-synuclein toxicity, *C. elegans*

## Abstract

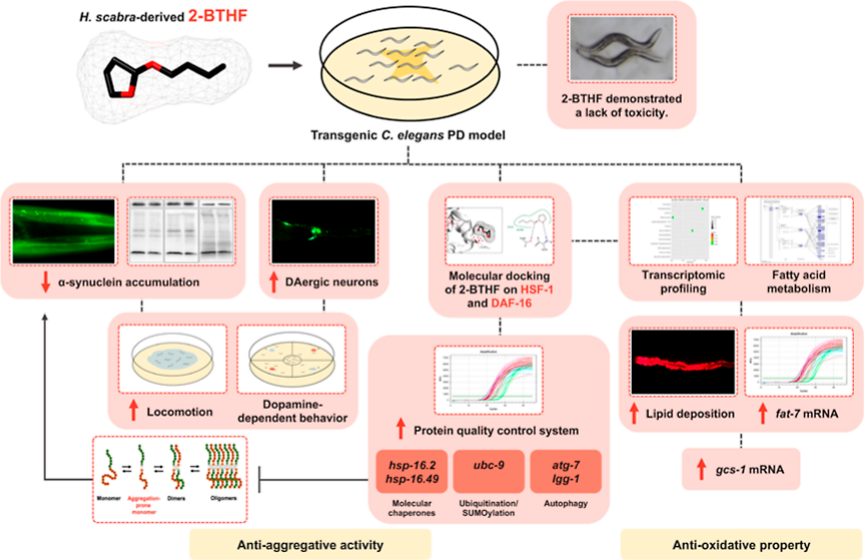

Aggregative α-synuclein and incurring oxidative stress are pivotal cascading events, leading to dopaminergic (DAergic) neuronal loss and contributing to clinical manifestations of Parkinson’s disease (PD). Our previous study demonstrated that 2-butoxytetrahydrofuran (2-BTHF), isolated from *Holothuria scabra* (*H. scabra*), could inhibit amyloid-β aggregation and its ensuing toxicity, which leads to Alzheimer’s disease. In the present study, we found that 2-BTHF also attenuated the aggregative and oxidative activities of α-synuclein and lessened its toxicity in a transgenic *Caenorhabditis elegans* (*C. elegans*) PD model. Such worms treated with 100 μM of 2-BTHF showed substantial reductions in α-synuclein accumulation and DAergic neurodegeneration. Mechanistically, 2-BTHF, at this concentration, significantly decreased aggregation of monomeric α-synuclein and restored locomotion and dopamine-dependent behaviors. Molecular docking exhibited potential bindings of 2-BTHF to HSF-1 and DAF-16 transcription factors. Additionally, 2-BTHF significantly increased the mRNA transcripts of genes encoding proteins involved in proteostasis, including the molecular chaperones *hsp-16.2* and *hsp-16.49*, the ubiquitination/SUMOylation-related *ubc-9* gene, and the autophagy-related genes *atg-7* and *lgg-1*. Transcriptomic profiling revealed an additional mechanism of 2-BTHF in α-synuclein-expressing worms, which showed upregulation of PPAR signaling cascades that mediated fatty acid metabolism. 2-BTHF significantly restored lipid deposition, upregulated the *fat-7* gene, and enhanced *gcs-1*-mediated glutathione synthesis in the *C. elegans* PD model. Taken together, this study demonstrated that 2-BTHF could abrogate aggregative and oxidative properties of α-synuclein and attenuate its toxicity, thus providing a possible therapeutic application for the treatment of α-synuclein-induced PD.

## 1 Introduction

Proteotoxicity results from misfolded or damaged proteins being transformed into non-native configurations, causing their dysfunctions and negatively affecting cellular homeostasis.^1^ Furthermore, misfolded proteins can polymerize, leading to their aggregations that are the root causes of several neurodegenerative diseases, such as Parkinson’s disease (PD), Alzheimer’s disease (AD), amyotrophic lateral sclerosis, and Huntington’s disease.^2^ Among these, PD is the second most common neurodegenerative disorder worldwide, characterized by the degeneration of dopaminergic (DAergic) neurons due to the formation of α-synuclein aggregation in the forms of Lewy bodies within these neurons.^3^ These aggregates progressively develop into insoluble filamentous polymers that accumulate in the nucleus, cytoplasm, and organelles, leading to alterations in the synaptic network and ultimately resulting in neuronal loss, thereby contributing to the clinical symptoms observed in PD.^4,5^ Physiologically, the normal functioning of eukaryotic cells requires protein quality control mechanisms that maintain proteostasis.^6^ These mechanisms include molecular chaperones, the unfolded protein response, the ubiquitin-proteasome pathway, and the autophagy-lysosomal system.^7^ They collectively serve to protect against the toxicity of protein aggregation and facilitate the degradation of the beyond-rescued misfolded proteins.^7^ The disruption of proteostasis can invariably lead to neuronal impairment.^7^ Additionally, α-synuclein accumulation has been identified as a prominent generator of oxidative stress, specifically lipid peroxidation, within primary cocultures of neurons and astrocytes.^8^ Consequently, both α-synuclein aggregation and oxidative stress are the two most damaging processes in PD patients. Understanding their interplay is essential for developing therapeutic strategies to mitigate synucleinopathies.

*Holothuria scabra* (*H. scabra*) is a species of sea cucumber, an invertebrate marine animal mostly found in the Indo-Pacific region. Sea cucumber-derived bioactive compounds have been reported to exhibit promising medical potentials, including antihyperlipidemic, anticancer, anti-inflammation, antioxidant, antihypertension, anticoagulant, immunomodulatory, and neuroprotective treatments.^9^ The beneficial compounds in sea cucumbers are reported to include fucosylated chondroitin sulfate, triterpene glycoside (saponins), glycosaminoglycan, phospholipids, peptides, phosphatidylcholines, collagen, phenols, amino acids, calcium, omega-6, and omega-9.^9^ These constituents and their health-promoting actions render sea cucumbers being a potential target for intense biomedical research as well as for food in the Asian region.^9^ Our previous studies have demonstrated that the small cyclic ether compound, 2-butoxytetrahydrofuran (2-BTHF), isolated from *H. scabra*, could abrogate amyloid-β (Aβ) oligomers through heat shock factor-1 (HSF-1)-mediated autophagy, consequently delaying paralysis in a *Caenorhabditis elegans* (*C. elegans*) model of AD.^10^ In addition, 2-BTHF has been found to extend lifespan and activate stress resistance through the DAF-16/FOXO and SKN-1/Nrf-2 signaling cascades in an aging *C. elegans* model.^11^ These findings indicate the potential therapeutic effects of 2-BTHF against AD and other neurological diseases. However, it remains unexplored whether 2-BTHF can similarly exhibit an anti-Parkinson effect. Hence, our present study aimed to investigate the anti-PD potential and the pertinent molecular mechanisms of 2-BTHF against α-synuclein-mediated toxicity in the transgenic *C. elegans* PD model.

*C. elegans* offers many advantages as a model system to study neurodegenerative diseases. The worms possess a short life cycle, a simple neuronal network, and a complete genome sequence that shares approximately 75% homology with mammals. Additionally, they can be easily cultured, and their nervous system pathway is evolutionarily conserved.^12^ The nervous system of *C. elegans* has been elucidated, revealing simple neuronal morphologies distinguished by uncomplicated processes.^13^ This includes the examination of neuron–glia interaction, cell migration and process development, transcription factors, and synaptic plasticity and function, as well as studies on the extracellular matrix.^13^ Nevertheless, *C. elegans* lacks an operational blood–brain barrier (BBB), allowing absorbed chemical molecules to rapidly diffuse into the nervous system.^14^ Consequently, these nematodes may be well-suited for studying a range of phenotypic and biochemical deficits observed in PD, such as age-dependent aggregation, impaired movement, DAergic neuron loss, disruption of dopamine-related behaviors, and heightened sensitivity to stress.^15^ In our study, we utilized two transgenic *C. elegans* strains, including NL5901 and UA44. The NL5901 strain (*unc-54p::yfp::*α*-syn*) expressed human α-synuclein in muscle cells, while the UA44 strain (*dat-1p::*α*-syn + dat-1p::gfp*) carried human α-synuclein gene expressed in DAergic neurons. We hypothesized that 2-BTHF, isolated from *H. scabra*, may mitigate the toxic aggregation of α-synuclein and reduce its oxidative action in these transgenic worms. In support of this hypothesis, we demonstrated the capacity of 2-BTHF in diminishing α-synuclein accumulation, augmenting antioxidation activity, stimulating protein quality control systems, reinstating lipid deposition, restoring DAergic neurons, and rescuing the worms from PD-related behaviors.

## 2 Results and Discussion

### 2.1 Low and Medium Doses of 2-BTHF Were Not Toxic to *C. elegans* NL5901 and UA44 Strains

**Figure 1:**
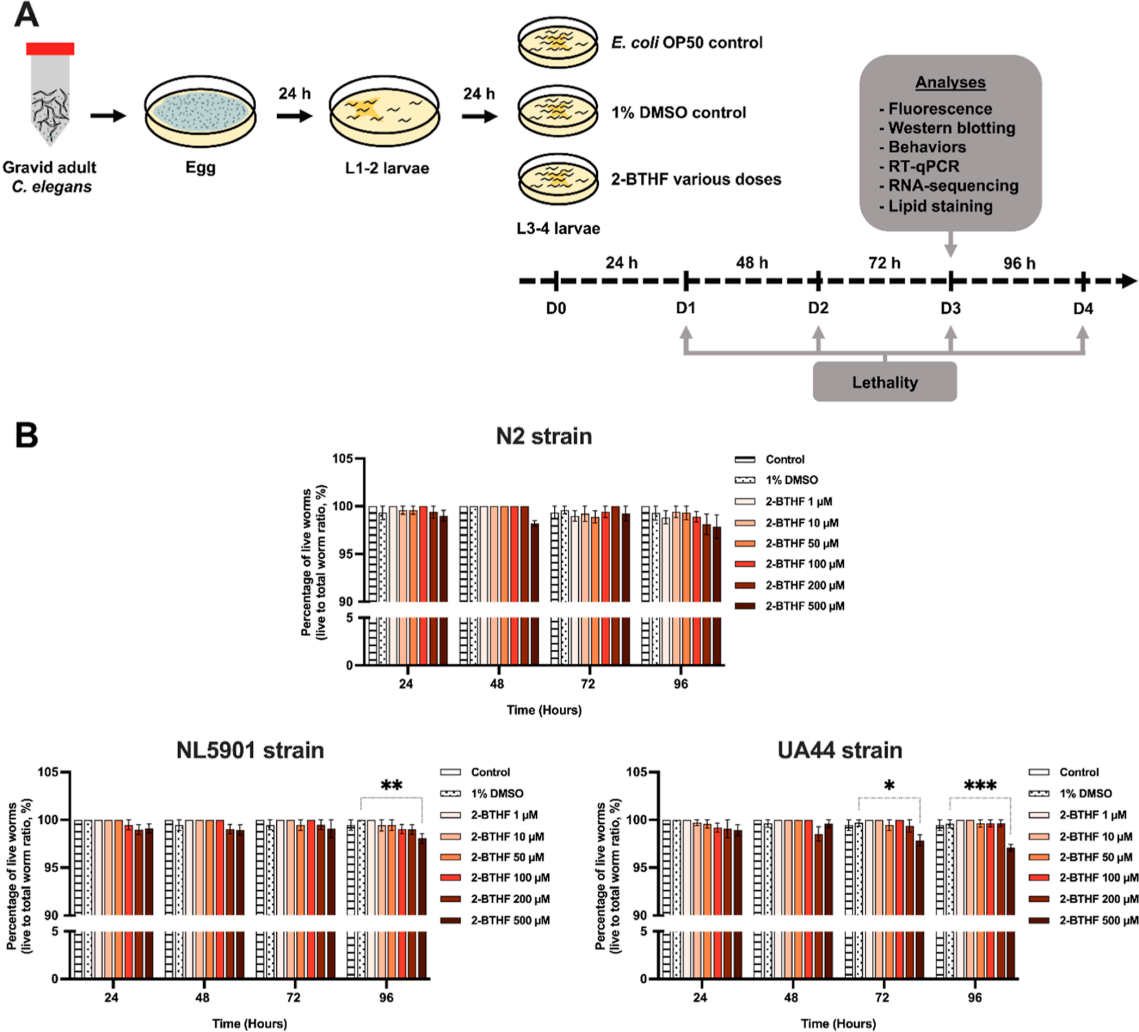
Worms experienced toxicity only when being exposed to a high dose of 2-BTHF at 500 μM. (A) Scheme of 2-BTHF treatment of the worms. (B) The percentage of live worms was assessed at 24 to 96 h for each dose of 2-BTHF. The results represent the mean ± SD of three independent experiments. A two-way analysis of variance (ANOVA) was employed, complemented by Bonferroni’s multiple comparison test. Statistical significance is indicated as ***p < 0.001, **p < 0.01, and *p < 0.05 compared to the 1% DMSO control.

To examine the optimal doses of 2-BTHF, a lethality assay was performed. 2-BTHF was mixed with *Escherichia coli* OP50, resulting in final doses of 1, 10, 50, 100, 200, and 500 μM. Control worms were grown in 1% DMSO. The percentage of live worms in the *E. coli* OP50 and 1% DMSO control groups showed no significant difference. A previous study reported that DMSO at concentrations up to 2% had no toxicity on the lifespan of the N2 strain.^16^ The lifespan of NL5901 worms remained unaffected by the presence of α-synuclein aggregates.^17^ Therefore, this study used treatment with 1% DMSO as a control, which could be confidently regarded as safe in each treatment. For N2 worms, all doses of 2-BTHF (1–500 μM) did not result in a significant decrease in the percentage of live worms compared to the 1% DMSO control group. Similarly, in transgenic strains, doses up to 200 μM of 2-BTHF showed no toxicity, while the 500 μM dose resulted in a significant decrease in the proportion of live worms in NL5901 worms at 96 h (98.06 ± 0.52%, *p* < 0.01) as well as in UA44 worms at 72–96 h (97.85 ± 0.58%, *p* < 0.05 and 97.09 ± 0.33%, *p* < 0.001, respectively) (Figure 1B). Therefore, the results indicate that 2-BTHF derived from *H. scabra*, at doses up to 200 μM, exhibited no toxicity in any of the tested strains. This finding suggests that the doses up to 200 μM were suitable for use in subsequent experiments.

### 2.2 2-BTHF Diminished α-Synuclein Accumulation in Transgenic *C. elegans*

**Figure 2:**
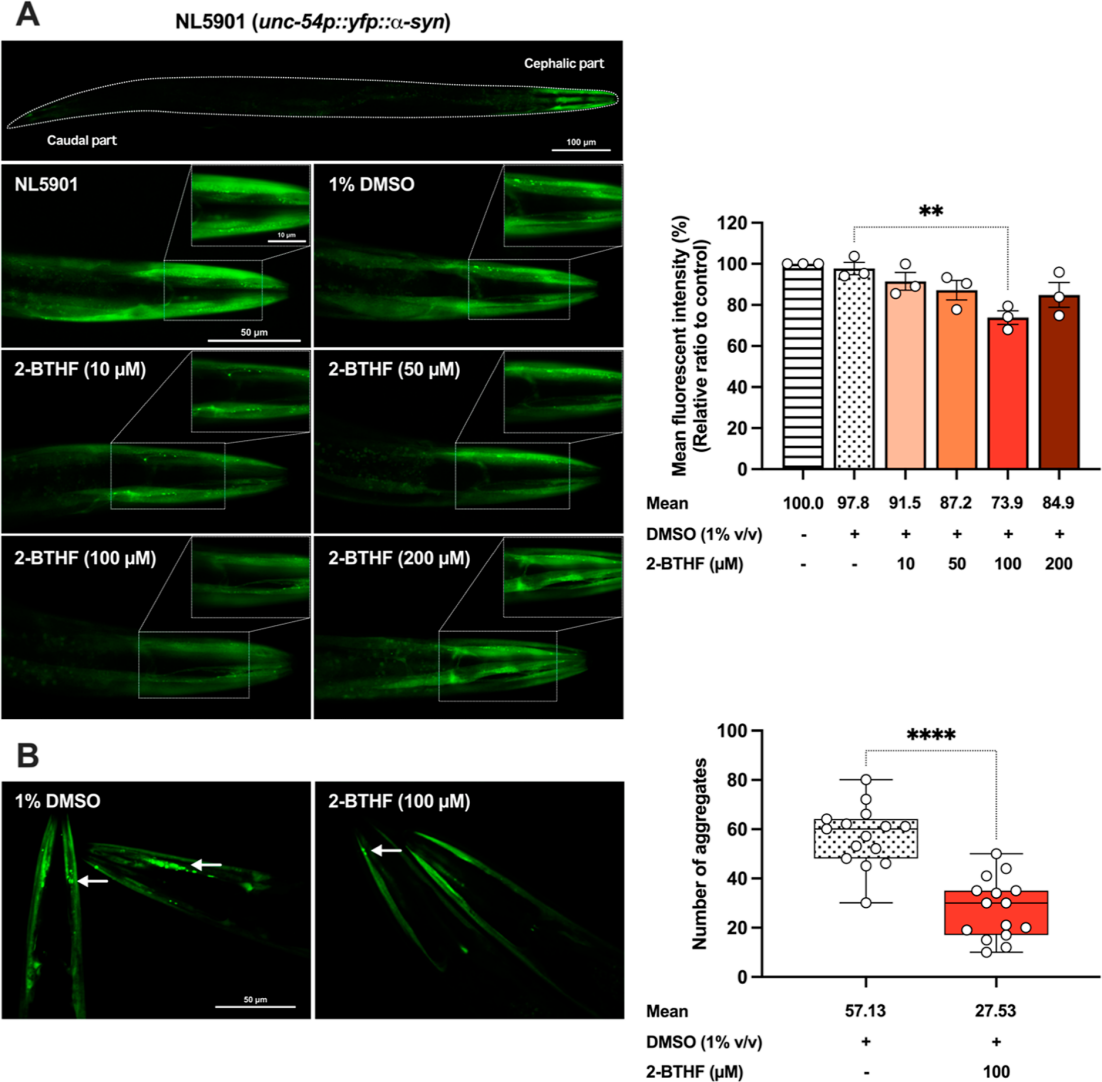
2-BTHF mitigated α-synuclein accumulation in NL5901 strain. (A) Fluorescence image and intensity of each whole worm were analyzed and representative images taken to depict YFP-tagged α-synuclein in the body wall muscle cells of NL5901 worms in the 1% DMSO- treated control group and in 2-BTHF-treated groups at various doses. The data represented the mean ± SEM of three independent experiments, with *n* = 30 worms per group. A one-way ANOVA was applied, followed by Dunnett’s multiple comparison test. (B) The quantification of α-synuclein aggregates in confocal images was performed by counting the number of fluorescence foci. The results, presented as the mean ± SD from three independent experiments, with *n* = 5 worms per group, were analyzed using a two-tailed paired *t*-test. Statistical significance is denoted as ****p < 0.0001 and **p < 0.01, indicating a significant difference compared to the untreated control.

The aggregation of human α-synuclein resulting from the overexpression of *SNCA* is a pathological characteristic of PD.^18^ To investigate the impact of 2-BTHF on α-synuclein accumulation, NL5901 worms were subjected to various doses of 2-BTHF, while a control group treated with 1% DMSO was used as the untreated control. Treatment with 100 μM 2-BTHF significantly reduced the intensity of α-synuclein fluorescence to approximately 73.86 ± 3.31% (*p* < 0.01) compared to the untreated control. However, the other doses showed only a slight decrease (10 μM, 91.48 ± 4.34%; 50 μM, 87.19 ± 4.77%; and 200 μM, 84.90 ± 6.10%) compared to the 1% DMSO control (Figure 2A). In addition to assessing fluorescent intensity, we also quantified the accumulation of α-synuclein aggregates in the head region of the worms. The group treated with 1% DMSO exhibited a substantial number of fluorescence foci, representing α-synuclein aggregates, with an average of 57.13 ± 12.15 aggregates. Conversely, administration of 100 μM 2-BTHF led to a significant reduction in α-synuclein fluorescence foci, with an average of 27.53 ± 12.28 (*p* < 0.0001) (Figure 2B). Previous studies have demonstrated that *H. scabra*-derived 2-BTHF possesses protective attributes that hinder Aβ aggregation. Structurally, 2-BTHF has been identified as a heterocyclic tetrahydrofuran (THF) compound, specifically a cyclic ether.^10^ A high concentration of THF has also been reported in terpenoid research, demonstrating enhanced bioactivity, including anti-Aβ aggregation, antioxidant, and anti-inflammatory properties.^19,20^ Crown ethers, which are cyclic polyethers, have also been reported to inhibit amyloidosis and the formation of amyloid fibrils.^21^ Therefore, it is possible that the cyclic ether 2-BTHF at 100 μM could interfere with fibrils in α-synuclein, acting as an effective dose for attenuating α-synuclein accumulation in transgenic *C. elegans*.

### 2.3 2-BTHF Mitigated α-Synuclein-Induced DAergic Neurodegeneration

**Figure 3:**
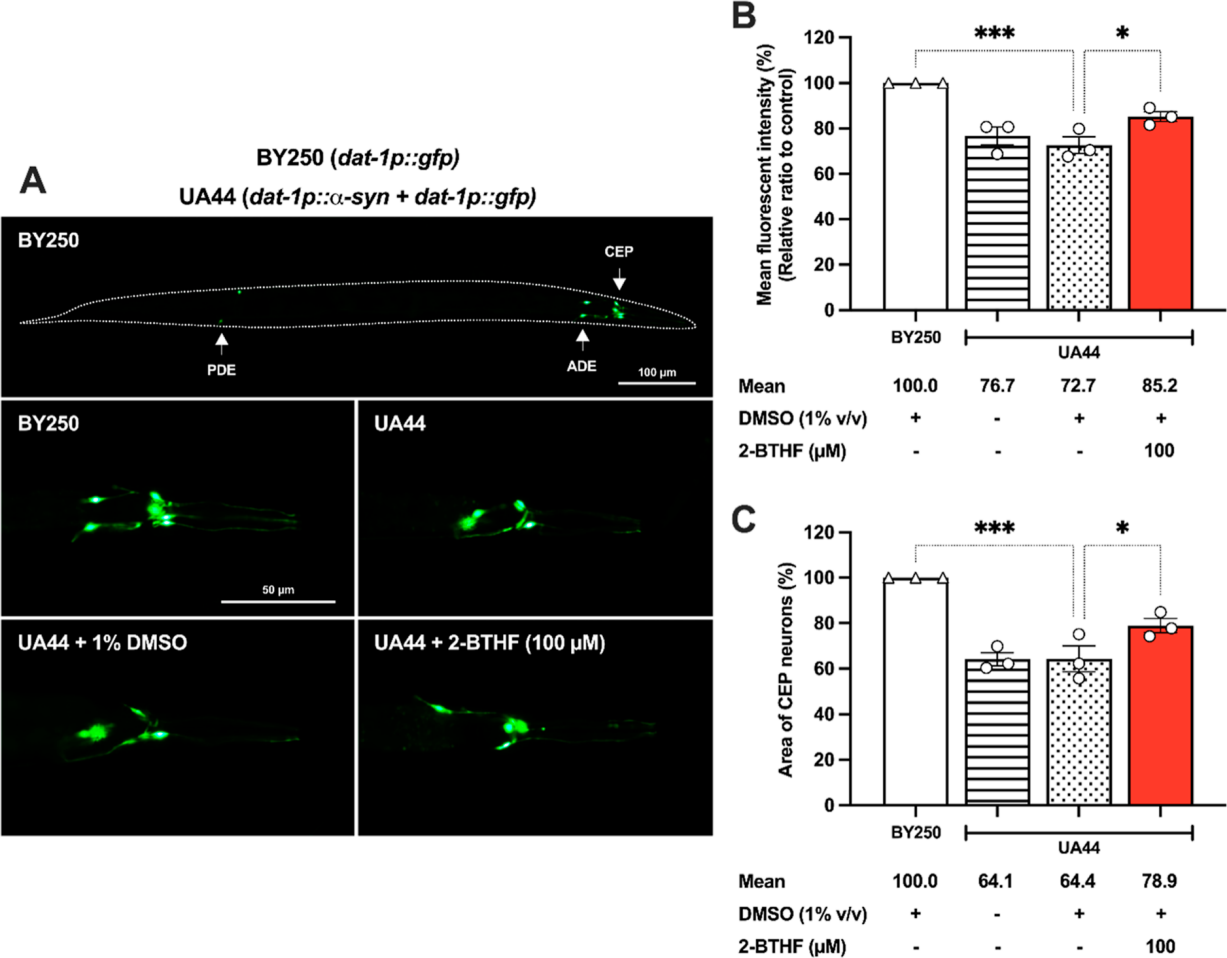
2-BTHF rescued DAergic neurons from α-synuclein-induced neuronal loss in UA44 *C. elegans*. (A) Fluorescence image and (B) intensity of CEP and ADE neurons showing α-synuclein and GFP in the DAergic neurons of UA44 worms in the 1% DMSO-treated control group and in 2-BTHF-treated groups. Normal control was established using the BY250 strain, which expressed GFP in the DAergic neurons. (C) The analysis involved the measurement of the area utilized by CEP neurons. The data represented the mean ± SEM from three separate experiments, each involving *n* = 30 worms per group. A one-way ANOVA was utilized, followed by Dunnett’s multiple comparison test. Statistical significance is denoted as ***p < 0.001 and *p < 0.05, indicating a significant difference compared to the untreated control.

The overexpression of α-synuclein inclusions disrupts cellular mechanisms leading to postmitotic differentiated human DAergic neuronal cell damage^22^ and impairs motor function, along with degeneration of DAergic neurons in the midbrain and striatum of monkeys.^23^ The hypothesis being proposed was that the reduction of α-synuclein accumulation could serve as a therapeutic target for PD. To prove this hypothesis, we examined the response to the treatment with 2-BTHF by UA44 worms which carry human α-synuclein specifically in the DAergic neurons. For a normal control, we utilized the BY250 strain, which expressed green fluorescent protein (GFP) in four cephalic (CEP) neurons along with their dendrites, as well as two anterior deirid (ADE) neurons and two posterior deirid (PDE) neurons (Figure 3A). The DMSO-treated UA44 worms exhibited a significant reduction in the fluorescence intensity of GFP-tagged DAergic neurons (72.65 ± 3.69%, *p* < 0.001). Remarkably, treatment with 100 μM 2-BTHF resulted in a significant increase in the DAergic neurons (85.21 ± 2.19%, *p* < 0.05) (Figure 3B). Furthermore, the fluorescent area of CEP neurons significantly decreased in DMSO-treated UA44 worms (64.38 ± 5.72%, *p* < 0.001), while 100 μM 2-BTHF preserved the CEP neuron area (78.94 ± 3.10%, *p* < 0.05) (Figure 3C). Previous studies have reported that a diterpene glycoside derived from *H. scabra* rescued DAergic neurons affected by α-synuclein-induced toxicity.^24^ Furthermore, *H. scabra* extract was found to decrease α-synuclein formation induced by MPP^+^-induced SH-SY5Y cell apoptosis through alterations in cellular metabolism, resulting in the enhancement of tyrosine hydroxylase (TH)-stained DAergic neurons.^25^ As a result, it is possible that 2-BTHF derived from *H. scabra* could alleviate α-synuclein-mediated toxicity, leading to an improvement in the GFP-tagged area of CEP DAergic neurons in the UA44 strain.

### 2.4 2-BTHF Decreased the Aggregation-Prone Monomeric α-Synuclein Targeted by α-Syn33 in Transgenic *C. elegans*

**Figure 4:**
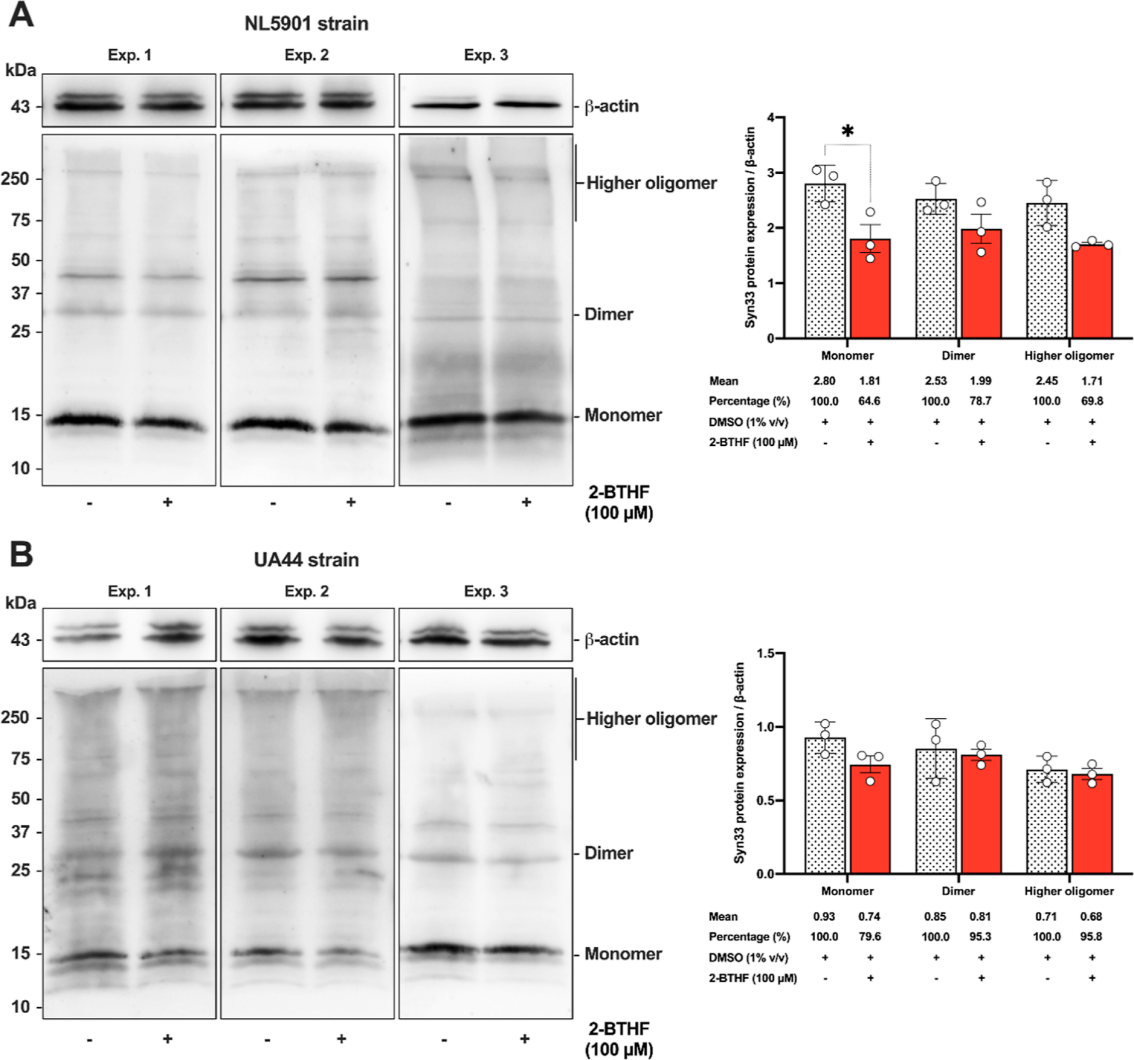
2-BTHF decreased the α-synuclein accumulation associated with the α-Syn 33 in transgenic *C. elegans*. (A) Western blot assay illustrated a reduction in α-synuclein accumulation in the NL5901 strain. (B) Western blot assay revealed a slight reduction in α-synuclein accumulation in the UA44 strain. The data represent the mean ± SD from three independent experiments (Exp.). A two-way ANOVA was performed, followed by Bonferroni’s multiple comparison test. Statistical significance is denoted as *p < 0.05, when compared to the untreated control.

To ascertain the decrease in fluorescence intensity in NL5901 worms treated with 2-BTHF, we conducted western blot analysis, employing staining with the α-Syn33 antibody to detect multimers, including the monomer (14.46 kDa), dimer (28 kDa), and higher oligomer (greater than 75 kDa). A positive control, β-actin (43 kDa) was employed to normalize the targeted protein expression levels. The 1% DMSO-treated worms served as a control group, and they exhibited no significant difference when compared to the normal *E. coli* OP50 control group (*p* = 0.1857) (Figure S1). The results revealed a significant decrease in the level of monomeric α-synuclein within muscle cells of NL5901 worms treated with 100 μM 2-BTHF (1.81 ± 0.43, *p* < 0.05) compared to the 1% DMSO-treated control (2.80 ± 0.34), with a reduction to approximately 64.6%, while dimeric and oligomeric α-synuclein showed a slight decrease, with no statistically significant difference (Figure 4A). In the UA44 strain, where α-synuclein is expressed in DAergic neurons, it was shown that 2-BTHF caused a slight reduction in monomeric α-synuclein, with no significant difference compared to the control (Figure 4B). In general, monomeric α-synuclein is characterized by three regions: the N-terminus (residues 1–60), the nonamyloid-β component (NAC, residues 61–95), and the C-terminus (residues 96–140). A previous study has reported that the N-terminus of α-synuclein possesses the capacity to modulate its aggregation, involving cross-linking specific residues and attracting binding proteins to the N-terminus. This modulation results in variations in aggregation kinetics.^26^ Therefore, focusing on the N-terminus, such as with α-Syn33 specific to residue 33, may be considered appropriate for identifying the aggregation form. The initial events in the misfolding mechanism are believed to influence the formation of amorphous aggregates of monomers.^26^ As such, misfolded α-synuclein at the monomeric level is proposed as a driving force for the aggregation-prone monomer, thereby facilitating the assembly of aggregates that eventually evolve into fibril formation.^27^ Based on the confocal analysis revealing the presence of α-synuclein aggregate foci in NL5901 worms, while higher oligomers are less prominent in western blot results, it is possible that amorphous aggregates may have decreased in size by the adult stage on day 3 of the worms. A previous study has reported an association between the induction of misfolded α-synuclein and toxicity induced by MPP^+^, leading to an increase in α-synuclein monomers. In contrast, treatment with a marine-derived xyloketal compound demonstrated efficacy in scavenging misfolded α-synuclein in the *C. elegans* model.^28^ Consequently, it is possible that 2-BTHF could interfere with the early misfolding and aggregation pathways, ultimately decreasing the aggregation-prone monomer of α-synuclein in transgenic NL5901 *C. elegans*. However, in the UA44 strain, it is possible that 2-BTHF may not mitigate the aggregation-prone nature of monomeric α-synuclein. This might be attributed to the transgenic expression of α-synuclein in DAergic neurons of the UA44 strain, resulting in lower levels of α-synuclein protein compared to the body muscle cells of NL5901 worms. Consequently, it is plausible that the α-synuclein protein profile is more readily detectable in NL5901 worms than in UA44 worms.

### 2.5 2-BTHF Exerted a Protective Effect against α-Synuclein-Induced Motility Deficit in NL5901 Worms and Restored Dopamine-Related Behavior in UA44 Worms

**Figure 5:**
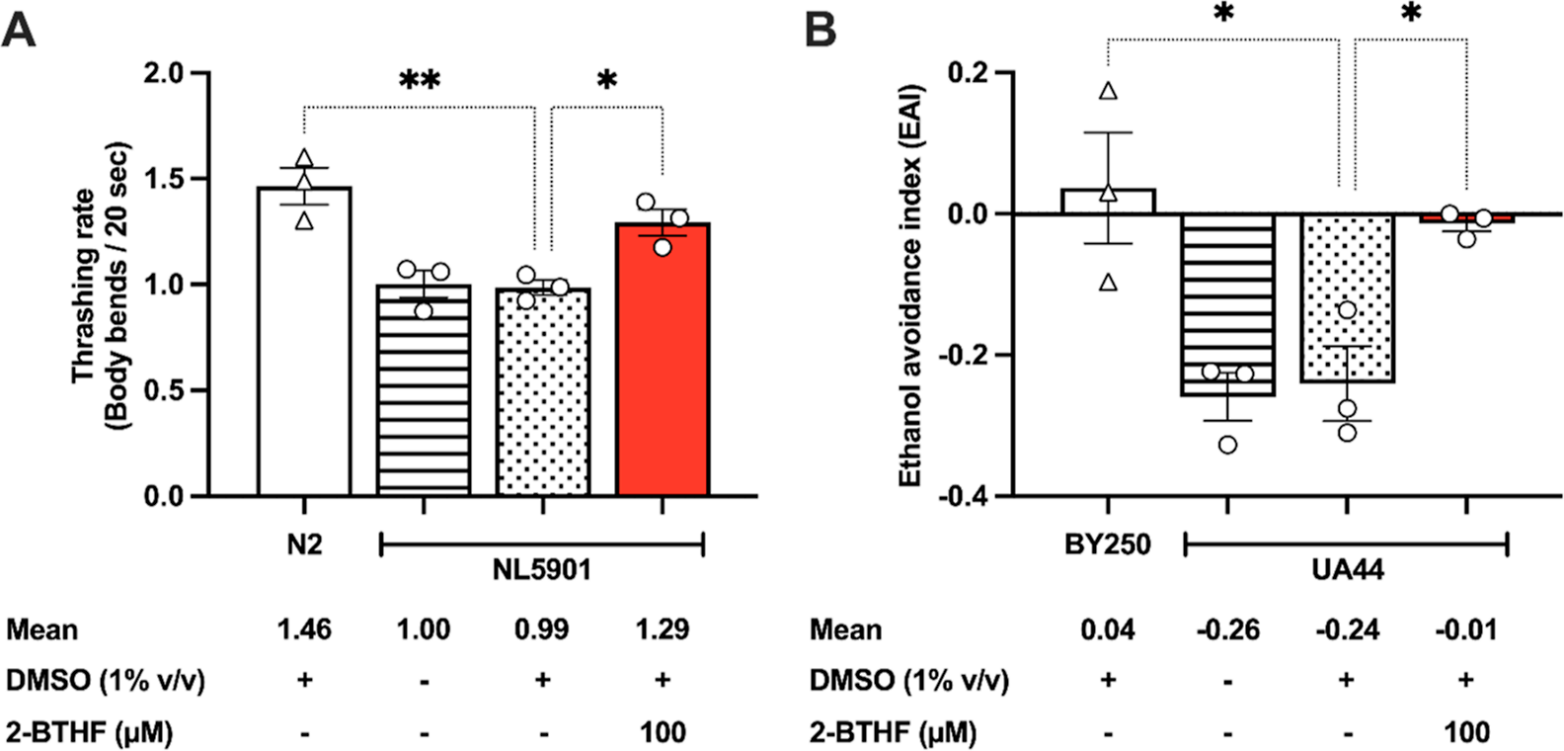
2-BTHF was effective in reversing the locomotion deficits and reinstating the dopamine-dependent behaviors. (A) The thrashing rates of N2 wild-type, NL5901, and NL5901 worms treated with 2-BTHF were analyzed using the wrMTrck plugin, and the results are presented in a graphical format. (B) Ethanol avoidance index (EAI) of normal worms, UA44, and UA44 strain treated with 2-BTHF is presented graphically. The data represent the mean ± SEM obtained from three distinct and separate experiments. A one-way ANOVA was performed, and Dunnett’s multiple comparison test was conducted. Statistical significance is indicated as **p < 0.01 and *p < 0.05, denoting a significant difference compared to the 1% DMSO control.

In prior studies, it was established that elevated levels of α-synuclein within the muscle cells of *C. elegans* were associated with impaired locomotion.^29^ Similarly, in rodent models, such overexpression was shown to induce motor deficits and asymmetry.^30^ In this study, NL5901 worms exhibited a significant decrease in thrashing rate (0.99 ± 0.04, *p* < 0.01) compared to the normal N2 worms (1.46 ± 0.09). However, upon treatment with 100 μM 2-BTHF, NL5901 worms exhibited a significant increase in their body bending (1.29 ± 0.06, *p* < 0.05) compared to the untreated group (Figure 5A). As a result, the overexpressed α-synuclein could potentially impact motor functionality, while 100 μM 2-BTHF may play a role in reducing α-synuclein accumulation in the body wall of the NL5901 strain.

We additionally investigated how α-synuclein worms influence dopamine-dependent behaviors, such as ethanol avoidance.^31^ The UA44 worms, expressing α-synuclein in their DAergic neurons, were examined. Our study revealed that UA44 worms exhibited a significant decrease in the EAI (−0.24 ± 0.05, *p* < 0.05) compared to the normal BY250 strain (0.04 ± 0.08). In contrast, treatment with 100 μM 2-BTHF significantly restored the EAI (−0.01 ± 0.01, *p* < 0.05) (Figure 5B). The excessive expression of α-synuclein in rodent models has been reported to intricately link with the degeneration of axons in DAergic neurons, compensatory upregulation of striatal D2/3 receptors, and a decrease in dopamine levels within the brain.^30^ Furthermore, the presence of α-synuclein results in the repositioning of synaptic vesicles affected by dopamine, causing disturbances in dopamine homeostasis.^32^ This disruption is a contributing factor to the degeneration of DAergic neurons in the *C. elegans* model.^32^ The deficiency in dopamine signaling, resulting from the absence of TH, is characterized by impaired dopamine-dependent behaviors.^33^ Importantly, the *H. scabra* extract was demonstrated to boost TH,^25^ the rate-limiting enzyme in catecholamine biosynthesis. This enzyme catalyzes the hydroxylation of tyrosine to l-DOPA within the dopamine signaling pathway.^34^ Therefore, it is possible that 100 μM 2-BTHF could potentially restore dopamine metabolism disrupted by α-synuclein-induced toxicity in UA44 worms.

### 2.6 Molecular Docking Analysis Revealed the Interaction between 2-BTHF and the Transcription Factors HSF-1 and DAF-16

**Figure 6:**
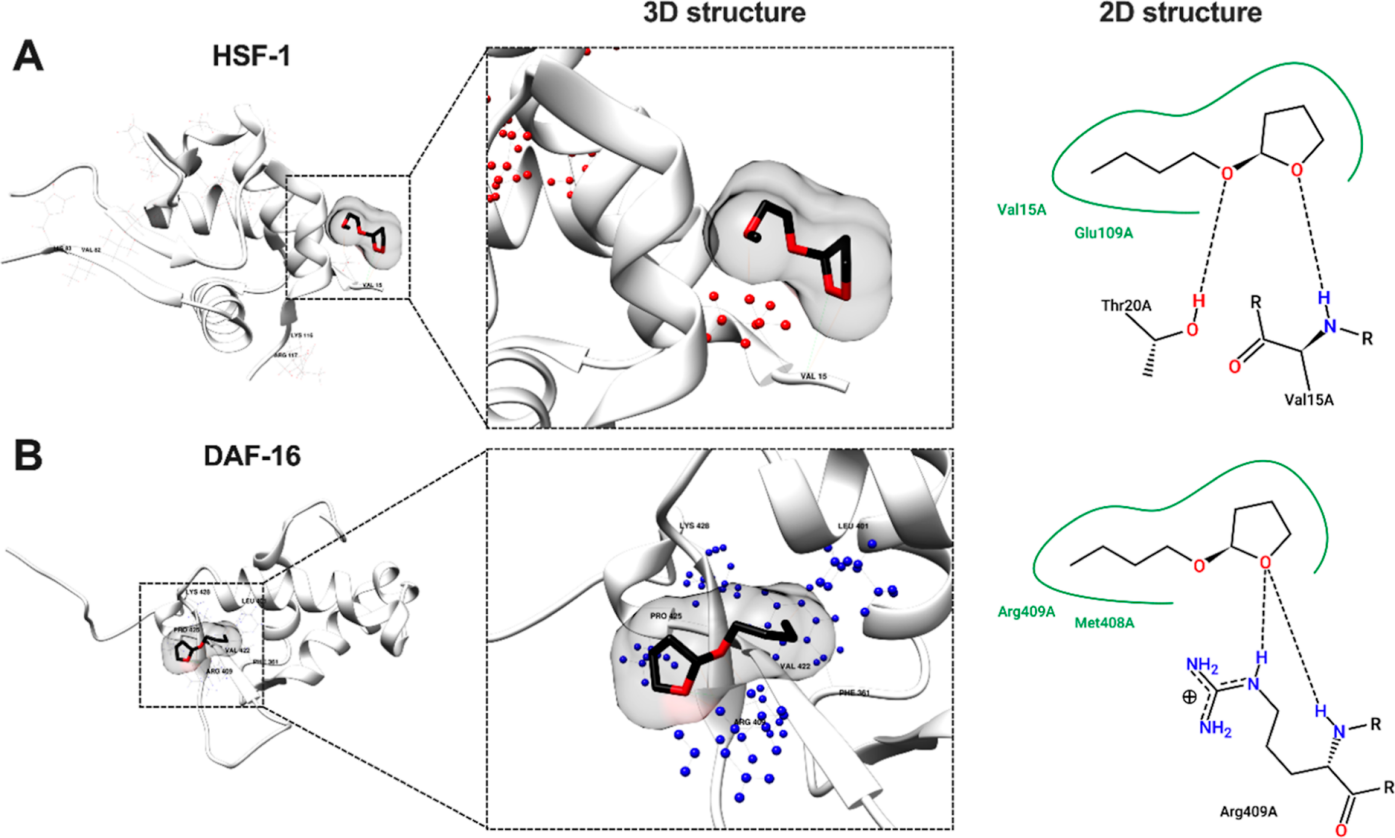
Postdocking analysis was conducted to evaluate the protein–ligand interaction. The interactions of 2-BTHF with the transcription factors (A) HSF-1 and (B) DAF-16 were visualized in three-dimensional (3D) structures using UCSF Chimera. Additionally, a two-dimensional (2D) structure illustrating the interactions between 2-BTHF and these transcription factors was generated with PoseView of ProteinsPlus. The structure indicates hydrogen bonds with dotted black lines and hydrophobic contacts with green lines.

Previous study indicated that *H. scabra* extracts have the potential to stimulate the insulin/IGF-1 signaling (IIS) pathway.^35^ This pathway relies on two essential transcription factors, HSF-1 and the FOXO transcription factor (DAF-16), resulting in the activation of downstream gene expression.^36^ Their collaboration has been demonstrated to delay protein aggregation and mitigate age-related diseases.^36^ Therefore, we hypothesized that 2-BTHF derived from *H. scabra* might initiate this pathway by interacting with these transcription factors. To validate this hypothesis, an initial step involved conducting molecular coupling analysis for prediction. To identify the docking of 2-BTHF with target proteins, we presented information on hydrogen-bonding pairs, hydrophobic bonds, binding energy scores, and additional detailed data in Table 1 and Figure S2. The results demonstrated that the oxygen atom within 2-BTHF formed hydrogen-bond interactions with the amino acid VAL 15 and THR 20, situated in the DNA-binding domain (DBD) of HSF-1, with an estimated binding affinity of −3.1 kcal/mol (Figure 6A). In the DAF-16 DBD, the oxygen atom of 2-BTHF formed a hydrogen bond with ARG 409, estimating a binding affinity of −4.2 kcal/mol (Figure 6B). Lower numerical values for the binding energy scores postdocking suggest a higher level of stability and a stronger binding affinity.^37^ Hydrogen-bonding interactions denote precise molecular recognition, a crucial factor in enhancing the stability of such interactions, thereby ensuring appropriate binding and optimal functional outcomes.^38^ Importantly, due to its molecular characteristics—weighing less than 400 Da and forming fewer than 8 hydrogen bonds—the small molecule 2-BTHF may potentially penetrate the BBB *via* free diffusion.^39^ As a result, these findings suggest that 2-BTHF has the potential to interact with HSF-1 and DAF-16 transcription factors, which could be appropriately applied in the treatment of neurodegeneration owing to its small molecular size.

**Table 1:** Molecular Coupling of 2-BTHF with the Transcription Factors

ligand	protein	interacting residues in		docking energy score (kcal/mol)
		hydrogen bond	hydrophobic contact	
2-BTHF	HSF-1	VAL 15, THR 20	VAL 15, GLU 109	–3.1
	DAF-16	ARG 409	MET 408, ARG 409	–4.2

### 2.7 2-BTHF Enhanced the Expressions of Genes Encoding Quality Control Systems in NL5901 *C. elegans*, *i.e.*, Molecular Chaperone, Ubiquitin-Conjugating Enzyme, and Autophagic Genes

**Figure 7:**
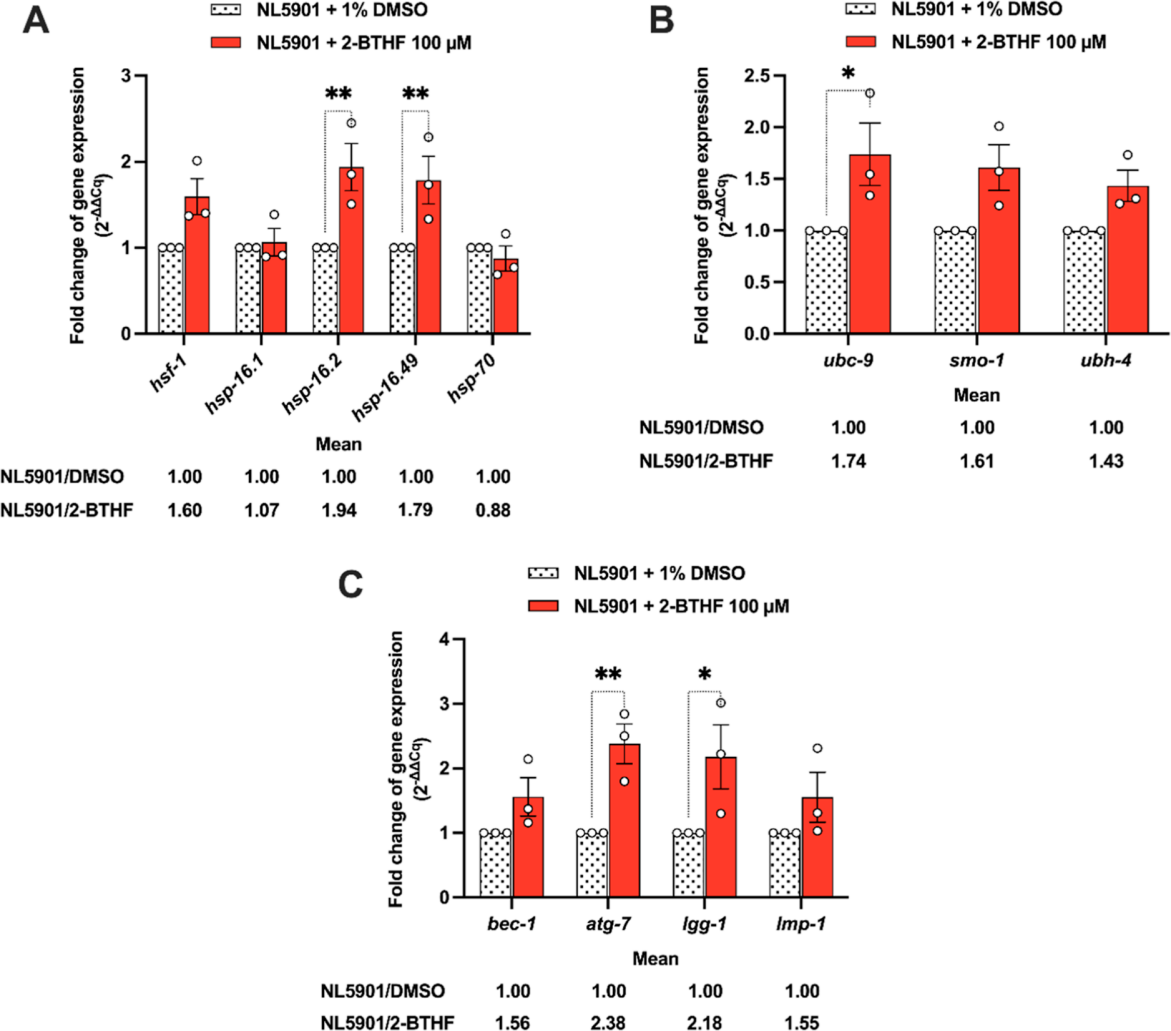
Impact of 2-BTHF on the expressions of genes encoding HSF-1, post-translational modification, and the autophagy-lysosomal pathway (ALP) in the NL5901 strain. mRNA levels corresponding to (A) HSF-1-dependent transactivation, (B) post-translational modification, and (C) ALP, which were quantified by reverse transcription-quantitative polymerase chain reaction (RT-qPCR). Relative gene expressions were normalized with the *act-1* gene as a control. The results represent the mean ± SEM obtained from three independent experiments. A two-way ANOVA was performed, followed by Bonferroni’s multiple comparison test. Statistical significance is indicated as **p < 0.01 and *p < 0.05 compared to the 1% DMSO.

The IIS pathways have been reported to collectively impact protein homeostasis, stress responsiveness, and longevity.^40^ This influence is mediated through the activity of key regulatory factors such as HSF-1, DAF-16, and molecular chaperones.^36,40^ These components collaborate to efficiently identify and manage misfolded and aggregation-prone proteins.^36,40^ Hence, our investigation focused on examining the expression of downstream genes regulated by HSF-1 and DAF-16 following treatment with 2-BTHF. Our results demonstrated that NL5901 worms treated with 100 μM 2-BTHF exhibited a significant upregulation of small heat shock proteins (HSPs), including *hsp-16.2* and *hsp-16.49*, with fold changes of 1.94 ± 0.27 (*p* < 0.01) and 1.79 ± 0.28 (*p* < 0.01), respectively (Figure 7A). In particular, the HSP27 homologue, *hsp-16* of *C. elegans*, exhibited an affinity for α-synuclein fibrils’ surface through mediation by both N- and C-terminal regions, leading to a reduction in their hydrophobic properties. This interaction revealed the potential to mitigate the cytotoxic effects associated with the aggregation of misfolded protein.^41^ Notably, the activation of *hsp-16* chaperones facilitates ATP-independent binding, effectively suppressing the aggregation pathway, particularly during the transition of misfolded proteins into small oligomers.^42^ Previous studies have indicated that the cointeraction of HSP-16.2 with Aβ entails the recognition of a conformational epitope linked to Aβ oligomerization, thereby mitigating Aβ toxicity *in vivo*.^43^ Furthermore, the activations of *hsp-16.11*, *hsp-16.2*, and *hsp-16.49* by palmatine exhibited neuroprotection in *C. elegans*, particularly with regard to the mitigation of Aβ-induced toxicity.^44^ Consequently, it is conceivable that small HSPs, particularly *hsp-16.2* and *hsp-16.49*, could potentially exert a significant influence in suppressing neurodegenerative diseases linked to the aggregation-prone proteins.

In addition, the activity of HSF-1 is regulated through dynamic post-translational modifications, including ubiquitination, SUMOylation, phosphorylation, and acetylation.^45^ HSF-1 undergoes both ubiquitination and SUMOylation mediated by ubiquitin-conjugating enzyme 9 (UBC9, known as *ubc-9* in *C. elegans*). This process further activates small ubiquitin-like protein modifier (SUMO, referred to *smo-1* in *C. elegans*) to attach misfolded proteins for degradation.^46^ To investigate the potential molecular mechanisms by which 2-BTHF activates the degradation system through ubiquitination/SUMOylation, we examined the mRNA levels of *ubc-9* and *smo-1* in NL5901 worms. The results revealed a significant upregulation of *ubc-9* with a fold change of 1.74 ± 0.30 (*p* < 0.05) (Figure 7B). Previous studies have highlighted the pivotal role of UBC9 in regulating SUMOylated α-synuclein.^47^ When UBC-9 is overexpressed, it could mitigate methamphetamine-induced α-synuclein aggregation through both the ubiquitin-proteasome system (UPS) and the ALP in both mice and SH-SY5Y models.^47^ Additionally, for other diseases like cardiovascular disease, UBC9 has demonstrated the capacity to reduce protein aggregates and preamyloid oligomers associated with proteotoxic CryAB^R120G^ by augmenting the functionality of the UPS.^48^ As a result, this study suggests that the upregulation of *ubc-9* may be involved in the early steps of dynamic ubiquitination and degradation pathways, particularly in UBC9 recruitment.^49^

The ALP functions as a mechanism for breaking down aberrant, accumulated, and aggregated macromolecules associated with HSF-1 activation.^50^ Therefore, we investigated the gene expression of the ALP following treatment with 2-BTHF. NL5901 worms treated with 100 μM 2-BTHF demonstrated a significant increase in the expression of *atg-7* (an E1-like enzyme involved in autophagosome activation) and *lgg-1* (an autophagosome marker) by a fold change of 2.38 ± 0.31 (*p* < 0.01) and 2.18 ± 0.50 (*p* < 0.05), respectively (Figure 7C). The THF derivative ANAVEX2-73, recognized as a Sigma-1 receptor agonist, has been reported to trigger the phosphorylation of ULK1. This process mediates the modulation of AMPK kinase and mTOR cascades, thereby instigating the formation of phagophores involved in canonical autophagic processes in HeLa cells.^51^ Additionally, ANAVEX2-73 has revealed effective facilitation of Aβ42 aggregate clearance *via* autophagy in nematodes.^51^ Considering these findings, it is possible that 2-BTHF might activate a similar mechanism. In another study, diterpene glycosides derived from *H. scabra* were found to enhance autophagic processes, including *bec-1* (involved in vesicular nucleation), *lgg-1*, and *atg-7*, in a *C. elegans* PD model. The study conducted RNA interference to specifically knock down these genes, with a notable reduction in the protective effects of *H. scabra* treatment, particularly targeting *lgg-1* and *atg-7*.^24^ This supports the role of ATG7 in facilitating the binding of phosphatidylethanolamine to LC3/LGG-1 during the lipidation process, a crucial step of degradative autophagy.^52^ Consequently, it is conceivable that 2-BTHF might enhance autophagy to eliminate aggregation-prone α-synuclein.

In conclusion, 2-BTHF has the potential to activate the protein quality control system against misfolding and aggregation-prone monomeric forms of α-synuclein. Then, we hypothesized whether 2-BTHF might also directly inhibit the aggregation of α-synuclein monomeric forms. To assess the interaction between 2-BTHF and the α-synuclein, molecular coupling analysis was employed, revealing that 2-BTHF can form a hydrogen bond with VAL 40 in the N-terminal region of the monomeric α-synuclein structure, indicating a binding affinity of −3.5 kcal/mol (Figure S3, Table S1). However, a more detailed investigation into this direct inhibition mechanism should be conducted in further studies.

### 2.8 RNA Sequencing Analysis Exhibited the Enriched Pathways in Transgenic NL5901 Worms Treated with 2-BTHF

**Figure 8:**
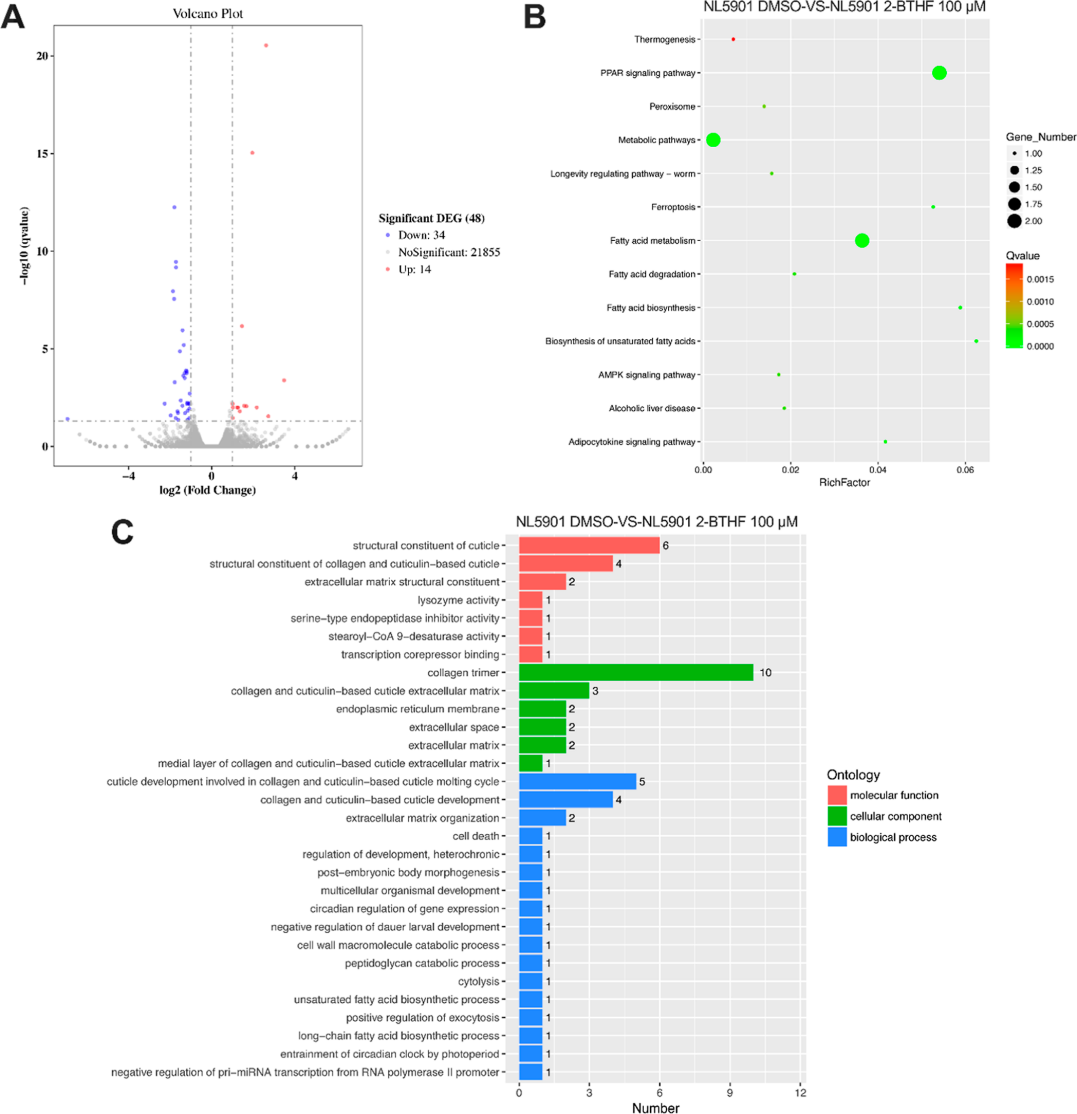
2-BTHF treatment impacts multiple metabolic pathways in NL5901 worms: (A) A volcano plot depicted the overall genes being regulated in 2-BTHF-treated worms, with some upregulated and others downregulated (*p* < 0.05). (B) The KEGG pathways included PPAR signaling, diverse metabolic pathways, thermogenesis, peroxisome function, regulation of longevity, ferroptosis, AMPK signaling, alcoholic liver disease, and adipocytokine signaling. (C) Gene ontology (GO) analysis revealed significant regulation of genes in 2-BTHF-treated worms across molecular function, cellular component, and biological process.

To investigate the dynamic response of cluster gene transcription following 2-BTHF treatment in NL5901 transgenic worms, we employed RNA sequencing (RNA-seq). The analysis indicated that treatment with 100 μM 2-BTHF resulted in the upregulation of 14 genes and downregulation of 27 genes in NL5901 worms (Figure 8A). Subsequent Kyoto Encyclopedia of Genes and Genomes (KEGG) pathway analysis identified significant involvement of various pathways, including PPAR signaling, various metabolic pathways, thermogenesis, peroxisome function, longevity regulation, ferroptosis, AMPK signaling, alcoholic liver disease, and adipocytokine signaling (Figure 8B). Notably, certain pathways, such as PPAR signaling, have been associated with processes related to α-synuclein misfolding and aggregation, oxidative stress, inflammation, and protein degradation regulation.^53^ Moreover, 2-BTHF predominantly targeted stearoyl-CoA desaturase 1 (SCD1) and acyl-CoA synthetase (ACS) genes in PPAR signaling cascades, central to fatty acid metabolism (Figure S4). GO analysis revealed the association of differentially expressed genes with molecular functions related to cuticle structure, cellular component mediating collagen trimer, and biological processes involving cuticle development (Figure 8C). Hence, these findings suggest that 100 μM 2-BTHF could effectively modulate various cellular metabolic pathways in NL5901 worms expressing α-synuclein, particularly fatty acid metabolism. Given that the accumulation of α-synuclein in PD brains may result in elevated levels of saturated fatty acids (SFAs, C16:0),^54^ it is plausible that 2-BTHF treatment could activate genes associated with lipid metabolism rather than other pathways, influencing cellular homeostasis.^54^ However, this study revealed a minor dynamic cluster of upregulated genes involved in the protein quality control system (Supporting Information). This phenomenon may be contingent upon normalization factors. Previous study highlighted the potential biases inherent in RNA-seq data analysis, especially regarding differential gene expression computation. Recognizing this, the study acknowledges the importance of computational suggestions for RT-qPCR validation. This validation was deemed valuable, particularly for genes susceptible to normalization biases based on factors such as transcript length, expression levels, and other unidentified parameters.^55^ Therefore, validation of the genes of interest would be confirmed through RT-qPCR. The expression levels of genes associated with the protein quality control system were previously demonstrated in Section 2.7.

### 2.9 2-BTHF Impacted on Lipid Deposition and the Antioxidant-Independent Mechanism in the Transgenic NL5901 Strain

**Figure 9:**
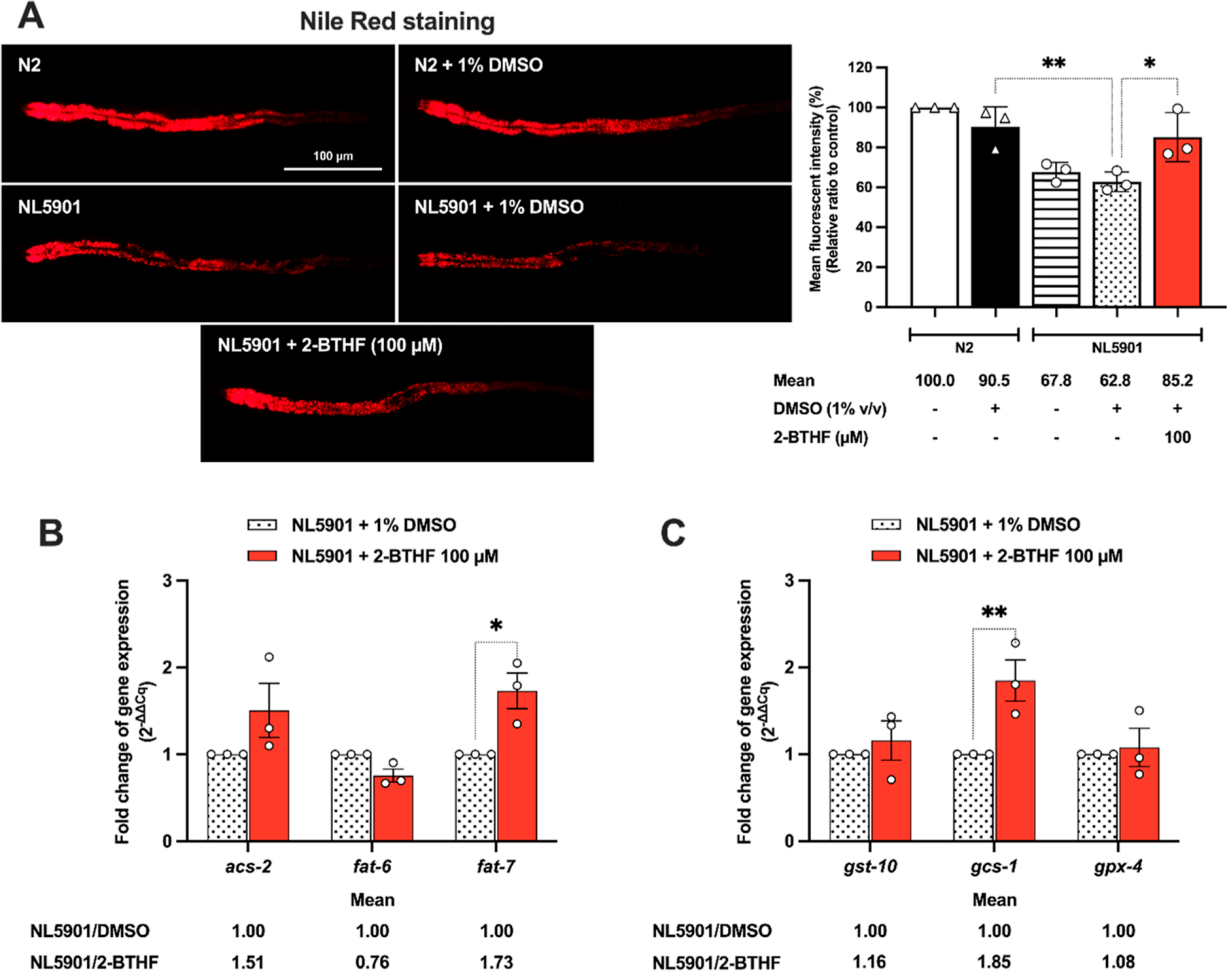
2-BTHF altered the lipid deposition and its metabolism in transgenic NL5901 worms. (A) Nile Red-stained lipid droplets in N2, NL5901, and NL5901 worms treated with 100 μM 2-BTHF were captured and presented as fluorescence intensity plotting in a graph. The N2 wild-type group served as a negative control. The findings illustrate the mean ± SEM derived from three independent experiments, with *n* = 30 worms per group. Data were compared by one-way ANOVA, followed by Dunnett’s multiple comparison test. mRNA levels of (B) fatty acid metabolism and the (C) glutathione (GSH) system were quantified through RT-qPCR. Relative gene expressions were normalized using the *act-1* gene as a control. The results represent the mean ± SEM derived from three independent experiments. Statistical analysis involved a two-way ANOVA, followed by Bonferroni’s multiple comparison test. Statistical significance is denoted as **p < 0.01 and *p < 0.05 when compared to the untreated group.

Alteration in lipid content and fatty acids is associated with PD.^56^ Moreover, RNA-seq analysis revealed modulation of lipid metabolism in 2-BTHF treatment. Thus, we assessed lipid content in worms expressing α-synuclein and those treated with 2-BTHF using Nile Red staining.^57^ The results indicated a significant reduction in lipid content in NL5901 worms (62.80 ± 2.86%, *p* < 0.01) compared to N2 worms (90.48 ± 5.72%). However, treatment with 100 μM 2-BTHF led to a notable increase in lipid storage (85.22 ± 7.09%, *p* < 0.05) compared to 1% DMSO control (Figure 9A). Lipids exhibit specific binding capabilities with α-synuclein.^58^ The decrease in lipid content in worms expressing α-synuclein may be attributed to the disruption of lipid composition by α-synuclein-induced toxicity.^56^ This protein aggregate appears to elevate lipid peroxidation due to an excess of reactive oxygen species (ROS).^8^ Conversely, the presence of 100 μM 2-BTHF reduced α-synuclein accumulation and significantly boosted lipid abundance. Therefore, this effect may be associated with the α-synuclein-induced lipid peroxidation pathway.

To examine the impact of 2-BTHF on fatty acid alteration, we examined genes related to ACS (*acs-2*) and SCD1 (*fat-6* and *fat-7*) (Figure S4). The results showed that 100 μM 2-BTHF significantly upregulated the *fat-7* gene to 1.73 ± 0.21 (*p* < 0.05) in NL5901 worms (Figure 9B). The IIS pathway is known for its role in regulating lipid synthesis and oxidation. Upregulation of DAF-16/FOXO has been reported to activate the downstream target, the SCD enzyme group, contributing to the regulation of lipid composition.^59^ FAT-6 and FAT-7 are Δ9 desaturase SCD1 enzymes responsible for converting fully saturated stearic acid (C18:0) to monounsaturated oleic acid (OA, C18:1^Δ9^), a major monounsaturated fatty acid.^60^ These enzymes are crucial for maintaining the normal fatty acid composition, including lipid homeostasis and membrane composition.^61^ In the brain, OA is a major constituent of membrane phospholipids, particularly in myelin.^62^ Notably, OA has been shown to alleviate 7-ketocholesterol-induced auto-oxidation in microglial cells implicated in neurodegeneration, affecting lipid peroxidation products and plasma membrane fluidity, whereas also increasing lipid droplet accumulation by Nile Red staining.^63^ Moreover, the *C. elegansfat-7* gene has been identified to influence lipid storage by inhibiting β-oxidation, operating through a PPAR-α-independent regulatory pathway that promotes fat consumption and stimulates fat storage.^60,64^ It is conceivable that 2-BTHF might activate the *fat-7* SCD1 enzyme, leading to an increase in monounsaturated OA to mitigate lipid oxidation, ultimately resulting in improved neutral lipid content. However, a more detailed investigation of this mechanism should be validated in future studies.

Previous research has observed increased production of ROS in worms expressing Aβ aggregation,^10^ while the ROS induced by α-synuclein has been linked to elevated levels of lipid peroxidation and oxidation.^8^ In contrast, the inhibitory mechanism of ROS-mediated oxidation involves GSH, which covalently interacts with glutathione peroxidase 4 (GPX4).^65^ Consequently, the GSH salvage system was investigated. The results indicated a significant elevation in the *gcs-1* gene after 2-BTHF treatment (1.85 ± 0.24, *p* < 0.01) (Figure 9C). Previous studies have demonstrated that α-synuclein aggregates, transitioning from its monomeric to its soluble oligomeric state, cause a reduction in endogenous GSH and subsequent neuronal toxicity.^66^ The *gcs-1* gene, a glutamate–cysteine ligase involved in intracellular GSH biosynthesis, plays a critical role in protecting *C. elegans* from oxidative stress.^67^ A previous study has reported that 2-BTHF (1 μg/mL) exhibits antioxidative stress properties against ROS in *C. elegans* with Aβ toxicity.^10^ These findings suggest that 100 μM 2-BTHF not only has the potential to restore lipid composition *via* fatty acid metabolism but also indirectly activates GSH synthesis. This activation aids in defending against free radicals caused by α-synuclein-induced toxicity.

## 3 Methods

### 3.1 Isolation of 2-BTHF from *H. scabra*

*H. scabra* specimens were obtained from the Coastal Fisheries Research and Development Center, Prachuap Khiri Khan, Thailand. The body walls were then collected, cut into small pieces, and stored at −80 °C prior to freeze-drying. This sea cucumber handling protocol was conducted ethically and approved by the Mahidol University-Institute Animal Care and Use Committee (MU-IACUC; MUSC66-001-631). Spectral data and details of the purified compound 5 (BU-P2) as 2-BTHF were provided in our previous publication.^10,24,35,68^

### 3.2 *C. elegans* Strain, Maintenance, and Synchronization

*C. elegans* strains utilized in this study were sourced from the *Caenorhabditis* Genetics Center (CGC, Minneapolis, MN, USA) as follows: N2 Bristol (wild-type) and transgenic strain NL5901 (*unc-54p::yfp::*α*-syn*) containing a yellow fluorescent protein (YFP)-linked reporter for fluorescence observation of α-synuclein in body wall muscle cells. Strain UA44 (*dat-1p::*α*-syn + dat-1p::gfp*) expressing α-synuclein and GFP in the DAergic neurons was provided by the Caldwell Laboratory (University of Alabama, USA). Strain BY250 (*dat-1p::gfp*) expressing GFP in DAergic neuronal cell bodies and neurites was provided by Prof. Dr. Randy Blakely at Florida Atlantic University, USA. All procedures involving *C. elegans* were conducted following established protocols of MU-IACUC (MUSC66-001-631). The procedures of maintenance and synchronization are provided in the Supporting Information.

### 3.3 Information of 2-BTHF Treatment

Considering the molecular weight of 2-BTHF (144.21 g/mol), a 20 mg/mL 2-BTHF master stock solution in 100% DMSO corresponds to an approximate concentration of 139 mM. Working stock solutions were then prepared at concentrations of 1, 5, 10, and 20 mM in 100% DMSO. These working stock solutions were mixed directly with *E. coli* OP50 (1:100) to achieve final concentrations of 10, 50, 100, and 200 μM of 2-BTHF in 1% DMSO. Additionally, an untreated control group consisting of 1% DMSO with *E. coli* OP50 was included. The untreated and treated compound mixtures were plated onto NGM plates supplemented with 2′-deoxy-5-fluorouridine (FUDR, Sigma-Aldrich, MO, USA), which inhibits embryo hatching. After drying at 37 °C overnight, the untreated and treated plates were stored at 4 °C until use.^24^ L3 larvae were plated onto plates with or without 2-BTHF treatment and incubated at 20 °C for 72 h. Subsequently, each analysis was conducted (Figure 1A).^69^

### 3.4 Lethality Assay

To assess the toxic effect of 2-BTHF on all strains, L3 larvae (30–50 worms) of N2, NL5901, and UA44 strains were cultured on an NGM plate containing *E. coli* OP50 mixed with 2-BTHF (final concentrations: 1, 10, 50, 100, 200, and 500 μM). Untreated controls were supplemented with 1% DMSO. Live and dead worms were then counted under a stereomicroscope at 24, 48, 72, and 96 h. Live worms were characterized by free movement, while dead worms were identified by their lack of movement or body lysis resembling cuticle breakdown.^70^ The proportion of live worms (live to total worm ratio) was calculated for each dose at regular time intervals.^71^ The experiment was independently performed in triplicate.

### 3.5 Fluorescence Microscopic Imaging of α-Synuclein Accumulation

To visualize the level of α-synuclein accumulation, we analyzed the transgenic NL5901 strain. L3 larvae of NL5901 worms were placed on NGM-FUDR plates containing *E. coli* OP50, with or without 2-BTHF doses, and incubated for 72 h at 20 °C. Subsequently, the worms were washed three times with M9 buffer and anesthetized with sodium azide (30 mM) on a 2% agarose pad glass slide. Whole bodies of immobilized worms were captured using a Nikon epifluorescence microscope (Nikon Eclipse Ci-L, Nikon Corp., Tokyo, Japan). The experiment was independently performed in triplicate, and 30 worms were analyzed per group per experiment. The fluorescence intensity of YFP-tagged α-synuclein in the body wall muscle cells was semiquantified using ImageJ software (National Institute of Health, NIH, Bethesda, MD, USA).^69^

### 3.6 Confocal Microscopic Imaging

To quantify the α-synuclein aggregates, fluorescence foci were assessed using the confocal laser scanning FV1000 equipped with a 488 nm laser (FLUOVIEW, Olympus Corp., Tokyo, Japan). Images were captured at 40× magnification, 800 × 800 resolution, with a *Z*-stack slice thickness of 5 μm. *Z*-stacks were created along the CEP region, spanning from the mouth tip to behind the pharyngeal bulb.^72^ The composite image of the worms was compiled using ImageJ software (National Institute of Health, NIH, Bethesda, MD, USA), providing the count of aggregates using Find Maxima function.

### 3.7 Measurement of DAergic Neurodegeneration

To investigate the degeneration of DAergic neurons caused by α-synuclein, we employed the UA44 strain and used the BY250 strain as a control for normal worms. After treatment, the worms were washed with M9 buffer and transferred onto a solution of 30 mM sodium azide on a 2% agarose pad glass slide to immobilize them. The slide was then sealed with a coverslip. Subsequently, the CEP region of all worms was photographed using a Nikon epifluorescence microscope (Nikon Eclipse Ci-L, Nikon Corp., Tokyo, Japan). The fluorescence intensity of four CEP neurons and two ADE neurons was analyzed using ImageJ software (National Institute of Health, NIH, Bethesda, MD, USA).^73^ The measurement of the area of CEP neuronal bodies was additionally conducted using the same software.^74^ Each experiment was conducted independently in triplicate, with a total of 30 worms per group analyzed in each trial.

### 3.8 Formaldehyde Cross-Linking and Western Blotting Assay

The α-syn oligomers consist of noncovalently bound monomers that dissociate into monomers after boiling in SDS prior to loading in SDS-PAGE, and a reactive cross-linker, such as formaldehyde, can stabilize the α-syn multimers based on their molecular weight.^75^ The worms were exposed to 4% formaldehyde solution before undergoing lysis and homogenization, followed by the quantification of soluble proteins. The samples were then boiled in loading buffer at 95 °C for 5 min before conducting the western blot procedures. α-Synuclein was detected using the rabbit anti-α-syn, oligomer-specific Syn33 polyclonal antibody (1:1000, ABN2265, Sigma-Aldrich, St. Louis, MO, USA), while β-actin was detected using the mouse anti-α-smooth muscle actin antibody monoclonal clone 1A4 (1:3000, Sigma-Aldrich, St. Louis, MO, USA). Detection was done using goat antirabbit or antimouse IgG–peroxidase-conjugated H + L (1:5000, Sigma-Aldrich, St. Louis, MO, USA). Finally, the band was visualized using a chemiluminescent gel documentation system (Alliance Q9 mini). The comprehensive methodologies are provided in the Supporting Information. The mean density of α-synuclein bands was analyzed using ImageJ software (National Institute of Health, NIH, Bethesda, MD, USA). The representative data were obtained from independent experiments performed in at least triplicate.

### 3.9 Measurement of *C. elegans* Motility

The thrashing assay is frequently used for analyzing locomotor phenotypes.^76^ The measurement of the thrashing rate was performed between normal worms (N2) and α-synuclein-tagged worms (NL5901). After treatment, the worms were cleaned using M9 buffer and then transferred to a fresh NGM plate containing M9 buffer. The worms were incubated for 1 min before video recording their body bending for 20 s (approximately 500 frames). Then, the bend detection ratio (thrashing rate) was analyzed using the wrMTrck plugin of ImageJ software (National Institute of Health, NIH, Bethesda, MD, USA).

### 3.10 Assay of Ethanol Avoidance Behavior

The ethanol avoidance assay has functional implications in dopamine-dependent behaviors.^31^ Following treatment, 50–100 worms were thoroughly washed with M9 buffer until free from *E. coli* OP50 and then transferred to the inner circle of the plate assay, divided into four quadrants. The worms were incubated at 20 °C to facilitate locomotion for 30 min. Subsequent to the incubation period, the worms within each quadrant were enumerated. The detailed plate assay and the EAI are included in the Supporting Information. The experiments were conducted in triplicate.

### 3.11 *In Silico*: Molecular Coupling

The interaction between 2-BTHF and transcription factors of HSF-1 and DAF-16 was computationally analyzed using AutoDock Vina software within UCSF Chimera 1.17.3 (University of California, San Francisco, USA). This facilitated the docking of the protein–ligand complex, following established protocols.^77^ 3D complexes of 2-BTHF and crucial proteins were generated. Next, the 2D structure was automatically developed using PoseView software of ProteinsPlus (University of Hamburg, Hamburg, Germany).^78^ The detailed procedures of docking are provided in the Supporting Information.

### 3.12 RNA Isolation and RT-qPCR

Approximately 1000–3000 worms were washed with ultrapure water (type I), ensuring the removal of bacteria. The cleaned worms were then centrifuged, and the supernatant was discarded. Total RNA was extracted using an RNA extraction kit (RNeasy, Qiagen, Germany), and the extracted RNA was stored at −80 °C until further use. RNA concentration was assessed utilizing a NanoDrop 2000 spectrophotometer (Thermo Scientific, Waltham, MA, USA).^69^ The detailed RT-qPCR is provided in the Supporting Information.

### 3.13 RNA Sequencing and Analysis

RNA-seq was conducted from two groups: DMSO-treated NL5901 and DMSO-2-BTHF-treated NL5901 worms. Total RNA was extracted using the TRIzol reagent (Ambion, Life Technologies, NY, USA) according to the provided protocol. Subsequently, RNA concentration was measured using the NanoDrop 2000 spectrophotometer (Thermo Scientific, Waltham, MA, USA). RNA solution in GENEWIZ RNA stabilization tubes with a concentration exceeding 50 μg/mL was dried using the Savant SpeedVac DNA130 vacuum concentrator system (Thermo Scientific, Waltham, MA, USA) at 30 °C for 2 h. The resulting dried RNA stabilization tubes were submitted to GENEWIZ for library preparation. The detailed procedures of mRNA library construction, sequencing, and data analysis are provided in the Supporting Information.

### 3.14 Analysis of Lipid Depositions

Nile Red, a fluorescent dye, is utilized to stain intracellular lipid content in the worms, and the staining procedure was conducted as previously described.^57^ A stock solution of Nile Red (0.5 mg/mL in acetone) was prepared and combined with *E. coli* OP50 in a ratio of 1:250. Subsequently, L3 larvae were cultured on NGM/Nile Red/OP50 plates, either with or without 100 μM 2-BTHF, and then incubated at 20 °C for 72 h. The worms were subsequently washed and mounted onto agar pads containing 30 mM sodium azide for anesthesia. A Nikon epifluorescence microscope (Nikon Eclipse Ci-L, Nikon Corp., Tokyo, Japan) was employed to detect the intensity of lipid deposition. Lipid contents in the whole worm bodies were quantified using ImageJ software (National Institute of Health, NIH, Bethesda, MD, USA). The experiment was performed in triplicate, with 30 worms being analyzed per group in each experiment.

### 3.15 Statistical Analysis and Graph

Data analyses were conducted employing GraphPad Prism software 9 (GraphPad Software Inc.). Each experiment was meticulously performed at least in triplicate. To compare variations between the control and 2-BTHF-treated groups, a one-way ANOVA was executed, followed by Dunnett’s multiple comparison test. For group analysis, a two-way ANOVA was employed, complemented by Bonferroni’s multiple comparison test. To compare the two groups, the results were analyzed employing a two-tailed paired *t*-test. Statistical significance was ascertained with a threshold of *p*-values below 0.001, 0.01, and 0.05.

## 4 Conclusions

The compound 2-BTHF (100 μM), a cyclic ether derived from *H. scabra*, displayed potential in mitigating α-synuclein-induced toxicity. Notably, it appeared to diminish the formation of polymers of the aggregation-prone monomeric form of α-synuclein. Furthermore, 2-BTHF demonstrated the capacity to restore both locomotor function and dopamine-dependent behaviors. Molecular docking illustrated 2-BTHF putative interactions with HSF-1 and DAF-16 transcription factors. It is suggested that through bindings and activations with these factors, 2-BTHF upregulated crucial protein quality control mechanisms. This includes clearance cascades involving molecular chaperones, ubiquitination/SUMOylation, and autophagy, thus helping to mitigate α-synuclein-mediated toxicity. 2-BTHF additionally triggered multiple metabolic pathways, including fatty acid metabolism, an antioxidant-independent mechanism, particularly involving GSH synthesis, and the restoration of neutral lipid deposition. The small molecule, 2-BTHF compound, has the potential to alleviate the pathogenesis linked to α-synuclein toxicity-mediated PD in the *C. elegans* model. Nonetheless, it is essential to validate its effects in mammalian models. Subsequently, clinical trials in humans must be conducted before the drug can be considered for PD treatment, as it emerges as a promising candidate for a novel therapeutic.

## Data Availability

The data can be found within the published article and its accompanying Supporting Information. Upon request, the corresponding author can provide the additional data supporting the conclusions reached in this study.

## Supporting Information Available

Elaborate descriptions of methods: effect of 1% DMSO in comparison to normal *E. coli* OP50 control; docking outcomes for highly ranked poses of 2-BTHF with HSF-1 and DAF-16 target; interaction between 2-BTHF and α-synuclein; exploration of KEGG pathway analysis focusing on the PPAR signaling pathway-related fatty acid metabolism; docking results of the interaction between 2-BTHF and human α-synuclein; and primer sequences employed in this investigation (PDF)
The differential genes expressions of group NL5901 + 1% DMSO vs NL5901 + 100 μM 2-BTHF (XLSX)
