# Supplementary material for: 2-Butoxytetrahydrofuran, Isolated from Holothuria scabra, Attenuates Aggregative and Oxidative Properties of α-Synuclein and Alleviates Its Toxicity in a Transgenic Caenorhabditis elegans Model of Parkinson’s Disease
Source: ACS Chem Neurosci. 2024 May 10;15(11):2182–97. doi: 10.1021/acschemneuro.4c00008 (PMC11157484; doi:10.1021/acschemneuro.4c00008)
Supplement: Supplementary file 1 — cn4c00008_si_001.pdf [file cn4c00008_si_001.pdf]

Supporting Information

2-Butoxytetrahydrofuran, Isolated from *Holothuria scabra*, Attenuates Aggregative and Oxidative Properties of α-Synuclein and Alleviates its Toxicity in Transgenic *C. elegans* Model of Parkinson's Disease

*Sukrit Promtang [1], Tanatcha Sanguanphun [2], Pawanrat Chalorak [3], Laurence S. Pe [4], Nakorn Niamnont [5], Prasert Sobhon [2], and Krai Meemon [2,6]**

[1] *Molecular Medicine Program, Multidisciplinary Unit, Faculty of Science, Mahidol University, Ratchathewi, Bangkok, 10400, Thailand*

[2] *Department of Anatomy, Faculty of Science, Mahidol University, Ratchathewi, Bangkok, 10400, Thailand*

[3] *Department of Radiological Technology and Medical Physics, Faculty of Allied Health Sciences, Chulalongkorn University, Pathumwan, Bangkok, 10330, Thailand*

[4] *Research Center for Neuroscience, Institute of Molecular Biosciences, Mahidol University, Salaya, Nakhon Pathom, 73170, Thailand*

[5] *Department of Chemistry, Faculty of Science, King Mongkut's University of Technology Thonburi, Bang Mod, Bangkok, 10140, Thailand*

[6] *Center for Neuroscience, Faculty of Science, Mahidol University, Ratchathewi, Bangkok, 10400, Thailand*

*Corresponding author, e-mail: krai.mee@mahidol.ac.th; Tel.: +66-2201-5407

Table of Content

Descriptions of methods. S1-S4

Figure S1. Effect of 1% DMSO in comparison to normal *E. coli* OP50 control S5

Figure S2. Docking outcomes for highly ranked poses of 2-BTHF with HSF-1 and DAF-16 S6

Figure S3. The interaction between 2-BTHF and α-synuclein S7

Figure S4. The exploration of KEGG pathway analysis focusing on the PPAR signaling pathway related fatty acid metabolism S8

Table S1. Docking results of the interaction between 2-BTHF and human α-synuclein S8

Table S2. Primer sequences employed in this investigation S9-S10

METHODS

***C. elegans* strain, maintenance, and synchronization.** All *C. elegans* strains were cultured on nematode growth medium (NGM) agar plates, and the plates were maintained in a temperature-controlled incubator at 20 °C. Living *Escherichia coli* (*E. coli*) strain OP50, a food source for *C. elegans*, was plated to the surface of the NGM plate. To isolate synchronous eggs, gravid worms were bleached using a bleaching solution (12% *(v/v)* sodium hypochlorite and 10% *(v/v)* 1M sodium hydroxide) for 10 minutes. The egg pellets were then collected through centrifugation, washed three times with M9 buffer, and transferred onto the surface of NGM plates without a food source. The plates were then incubated at 20 °C, allowing the embryos to hatch and reach the L1 stage. To obtain L3 larvae, hatched L1 stage was transferred onto NGM with *E. coli* OP50 and incubated at 20 °C for 24 h [1].

Formaldehyde cross-linking and Western blotting assay. For formaldehyde cross-linking, a 4% paraformaldehyde solution in 1X PBS was incubated at 80 °C for 2 hours to obtain a formaldehyde solution for tissue fixation. Briefly, after 72 hours of treatment, the NL5901 strain was washed with M9 buffer and then resuspended in a 4% formaldehyde solution. The worms were incubated with mild agitation for 10 minutes at room temperature, followed by centrifugation. The supernatant was discarded, and the reaction was quenched three times with 1M Tris/PBS (pH 7.4). The worms were then lysed and homogenized by grinding with a pestle in lysis buffer (pH 7.4) containing 100 mM *(w/v)* Tris-HCl, 10 mM *(w/v)* EDTA, 1.5 mM *(w/v)* NaCl, 1% *(v/v)* Triton X-100, 1% *(v/v)* glycerol, 1% *(v/v)* NP-40, 1% *(w/v)* SDS, 10 mM *(w/v)* $CaCl_2 \cdot 2H_2O$, 1 mM *(w/v)* PMSF, 1 mM *(w/v)* NaF, and 1% *(w/v)* protease inhibitor cocktail [2]. The lysate containing the soluble fraction was collected, and the total lysed protein was measured using the Pierce™ BCA Protein Assay Kit (23225, Thermo Fisher Scientific, Waltham, MA, USA).

The samples were boiled in loading buffer (125 mM *(w/v)* Tris-HCl pH 6.8, 4% *(w/v)* SDS, 20% *(v/v)* glycerol, 4% *(v/v)* β-mercaptoethanol, and 0.02% *(w/v)* bromophenol blue) at 95 °C for 5 minutes. Subsequently, protein samples (30 μg/lane) were loaded onto a 12.5% SDS-Tris-PAGE gel and run in a running buffer (100 volts, 80 mA, 2 hours), followed by transfer to a nitrocellulose membrane (0.45 μm, Whatman GmbH-Germany) in a transferring buffer (100 volts, 400 mA, 1.5 hours). To visualize the proteins, the blotted membrane was stained with ponceau s solution for 1 minute. Afterward, the blotted membrane was blocked with 5% bovine serum albumin (BSA) in TBS-Tween-20 for 1 hour, stained with primary and secondary antibodies, and incubated with ECL

chemiluminescence (ECL, Thermo Fisher Scientific, Waltham, MA, USA) to enhance the positive bands, followed by visualization using a chemiluminescent gel documentation system (Alliance Q9 mini).

Assay of ethanol avoidance behavior. The NGM plate used for this assay was divided into four quadrants as follows: top left (A), top right (B), bottom left (C), and bottom right (D). An inner circle with a radius of 0.5 was demarcated at the center for worm placement. The normal control quadrants (A and D) were supplemented with M9 buffer (50 μL), while the ethanol-exposed quadrants (B and C) were placed with approximately 50 μL of 100% ethanol. After counting the worms in each quadrant, the ethanol avoidance index (EAI) was computed using the equation: EAI = (number of worms in A and D quadrants) – (number of worms in B and C quadrants) divided by the total number of worms. Notably, the worms residing within the inner circle were excluded [1].

***In Silico*: Molecular coupling.** The molecular docking was performed using Autodock Vina software within UCSF Chimera 1.17.3 (University of California, San Francisco, USA). The crystal arrangements of heat shock factor1-DBD complex (PDB ID: 5HDN), the forkhead domain of DAF-16a (PDB ID: 2MBF), and human α-synuclein (PDB ID: 1XQ8) were obtained from the RCSB Protein Data Bank. The molecular 3D structure of 2-BTHF was sourced from PubChem (PubChem CID: 2724555). Initially, undesirable protein chains and ligands were eliminated. Subsequently, a surface/binding analysis was conducted on the protein chain receptor, followed by Dock Prep (solvent deletion, hydrogen addition, charge inclusion). Following this, precise docking of the 2-BTHF ligand with the protein chain receptor was executed using the Autodock Vina software integrated into Chimera. The grid box was set to cover the protein, and polar hydrogens, merge charges, and lone pairs removal were assigned to the models in the receptor and ligand options. The outcomes were evaluated based on a clash parameter involving Van der Waals (VDW) overlap and potential hydrogen (H)-bonding pairs within a range of 0.2 angstroms (Å). During the docking process, we selected lower-energy conformations for additional analysis (Figure S2, S3).

RNA isolation and RT-qPCR. RNA (2 μg) was converted into complementary DNA (cDNA) using iScript^{TM} Reverse Transcription Supermix (Bio-Rad, Hercules, CA, USA). The resulting cDNA was diluted with SsoFast EvaGreen Supermix with Low ROX qRT-PCR (Bio-Rad, Hercules, CA, USA) at a 1:10 ratio and combined with specific gene's forward and reverse primers (Table S2). Real-time PCR was conducted by initially holding the sample at 95 ºC for 30

seconds, followed by denaturation at 95 ºC for 5 seconds and annealing at 60 ºC for 30 seconds. This cycle was repeated 44 times, and afterward, the sample underwent melt curve analysis by heating it up to 95 ºC. The quantitative cycle (Cq) was determined as a result of this analysis. The Cq data of both the control and treated groups were calculated using the $2^{-(\Delta\Delta Cq)}$ equation, representing the relative fold change of each gene compared to the *act-1* control [1]. The experiments were conducted in at least triplicate.

RNA-sequencing (RNA-seq) and analysis. For library preparation, 1 μg of total RNA served as the subsequent library preparation process. Poly(A) mRNA enrichment was accomplished by using Oligo(dT) beads. The mRNA fragmentation through exposure to divalent cations at elevated temperatures, followed by random primer-based priming. Subsequently, the synthesis of first-strand cDNA and second-strand cDNA was conducted. Then, the purified double-stranded cDNA was treated to end-repair and dA-tailing in a unified reaction, succeeded by T-A ligation to attach adaptors to both ends. Size selection of adaptor-ligated DNA was carried out using DNA Clean Beads. Each sample was then subjected to PCR amplification with P5 and P7 primers, and the PCR products were subjected to validation. The resulting libraries, each differentiated by unique indices, were multiplexed and loaded onto an Illumina HiSeq/ Illumina Novaseq/ MGI2000 instrument for sequencing. The sequencing was performed in a 2x150 paired-end (PE) configuration in accordance with the manufacturer's instructions.

For data analysis, **quality control:** To produce high-quality clean data, the fastq-formatted pass filter data underwent processing via Cutadapt (V1.9.1) to eliminate technical sequences like adapters, PCR primers, or their fragments, alongside base qualities below 20 (phred cutoff: 20). The parameters used for this processing included an error rate of 0.1, an adapter overlap of 1 base pair, a minimum length requirement of 75, and a threshold of 0.1 for the proportion of ambiguous bases (N).

Alignment: Initially, reference genome sequences and gene model annotation files of the pertinent species were acquired from genome databases, such as UCSC, NCBI (availabe at, https://www.ncbi.nlm.nih.gov/datasets/genome/GCF_000002985.6/), and ENSEMBL. Subsequently, the reference genome sequence was indexed using Hisat2 (V2.2.1), followed by aligning the clean data to the reference genome using the Hisat2 software (V2.2.1).

Expression analysis: Initially, transcripts in fasta format were generated from a known gff annotation file and appropriately indexed. Subsequently, using this file as a reference gene file, HTSeq (V0.6.1) estimated the levels of gene and isoform expression from the pair-end clean data.

Differential expression analysis: The analysis for differential expression employed the DESeq2 Bioconductor package (V1.6.3), which utilizes a model grounded on the negative binomial distribution. The estimates for dispersion and logarithmic fold changes integrate data-informed prior distributions. The adjusted *p*-values (Padj) of genes were established at a threshold of <= 0.05 to identify differentially expressed genes (DEGs).

GO analysis: GOSeq (V1.34.1) was utilized to identify Gene Ontology (GO) terms annotating a list of enriched genes with a significant adjusted *p*-value (padj) less than or equal to 0.05. Additionally, topGO (V2.18.0) was employed to visualize Directed Acyclic Graphs (DAG).

KEGG enrichment analysis: KEGG (Kyoto Encyclopedia of Genes and Genomes) represents a comprehensive database encompassing genomes, biological pathways, diseases, drugs, and chemical substances (source: http://en.wikipedia.org/wiki/KEGG). Internal scripts were developed to enrich significantly differentially expressed genes in KEGG pathways.

RESULTS

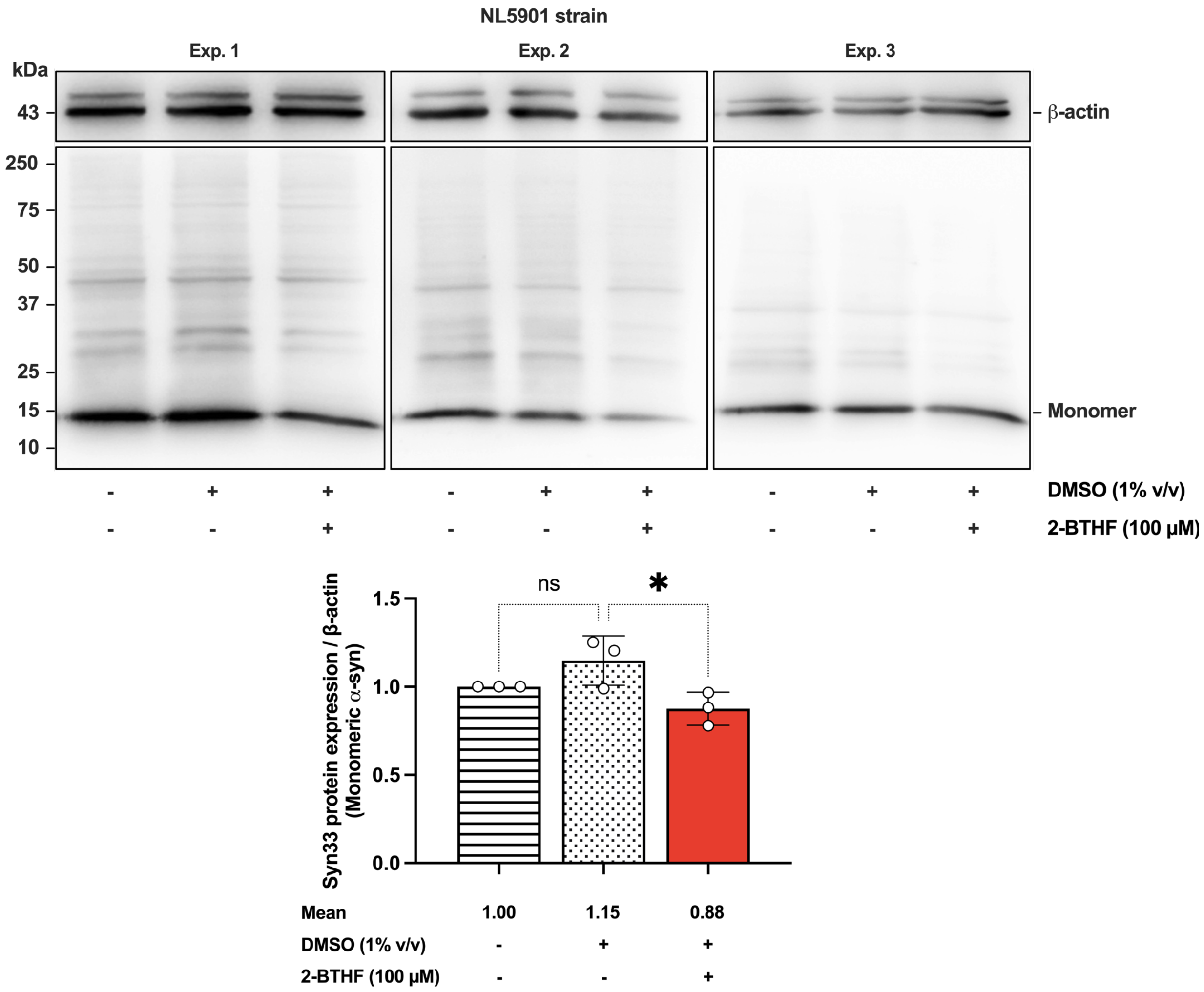

Figure S1. 2-BTHF diminished the accumulation of monomeric α-synuclein linked to α-Syn 33 in transgenic NL5901 *C. elegans*, while 1% DMSO yielded non-significant results (ns) when compared to the normal *E. coli* OP50 control. The data is presented as the mean±SD obtained from three independent experiments (Exp.). A one-way ANOVA was calculated, followed by a Dunnett's multiple comparison test. Statistical significance is denoted as $*p<0.05$ in comparison to the 1% DMSO untreated control; ns, non-significance.

A **HSF-1**

Mode	Binding affinity (kcal/mol)	Dist from best mode	
		rmsd* l.b.	rmsd* u.b.
**1**	**-3.1**	**0.000**	**0.000**
2	-2.8	29.157	31.102
3	-2.8	10.428	11.651
4	-2.7	9.824	11.023
5	-2.6	12.186	13.436
6	-2.6	10.442	11.639
7	-2.3	10.097	11.279
8	-2.3	9.774	11.238
9	-2.2	9.221	10.629

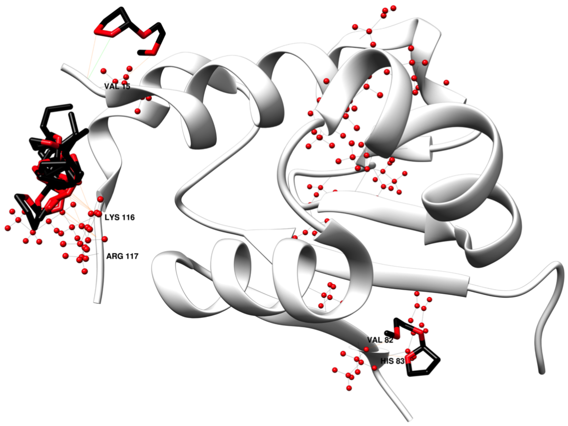

B **DAF-16**

Mode	Binding affinity (kcal/mol)	Dist from best mode	
		rmsd* l.b.	rmsd* u.b.
**1**	**-4.2**	**0.000**	**0.000**
2	-3.3	14.028	14.844
3	-3.2	13.043	13.923
4	-2.9	14.001	14.755
5	-2.4	12.078	13.417
6	-1.9	18.613	19.518
7	-1.9	19.246	20.545
8	-1.8	13.986	15.420
9	-1.7	14.942	15.677

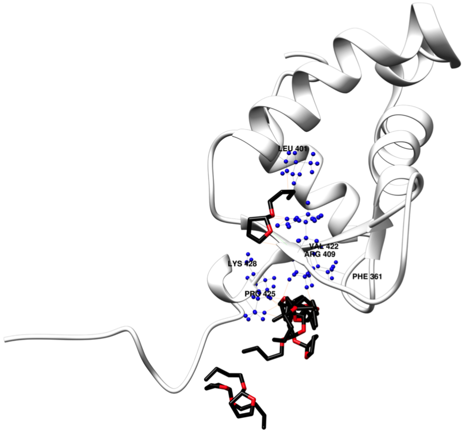

Figure S2. Docking results of 2-BTHF were analyzed on the key protein targets: (A) HSF-1 and (B) DAF-16. The range of Root Mean Square Deviations (RMSDs) was determined. RMSD values were evaluated in relation to the optimal mode, focusing on mobile heavy atoms. The analysis included determination of both RMSD lower bound (rmsd* I.b.) and RMSD upper bound (rmsd* u.b.), accounting for whether atoms were matched during the distance computation. The image was generated to depict all docking configurations of 2-BTHF ligand onto the respective transcription proteins. The highly favorable binding conformation, highlighted in orange, was chosen to be representative of the high affinity interaction.

A

α-syn	Mode	Binding affinity (kcal/mol)	Dist from best mode	
			rmsd* l.b.	rmsd* u.b.
	1	-3.5	0.000	0.000
	2	-3.1	1.547	2.23
	3	-3.0	1.407	2.37
	4	-3.0	2.078	4.888
	5	-2.8	117.615	119.827
	6	-2.8	3.093	4.321
	7	-2.7	3.148	5.14
	8	-2.7	129.363	131.562
	9	-2.7	2.168	4.579
	10	-2.7	2.767	5.237

B

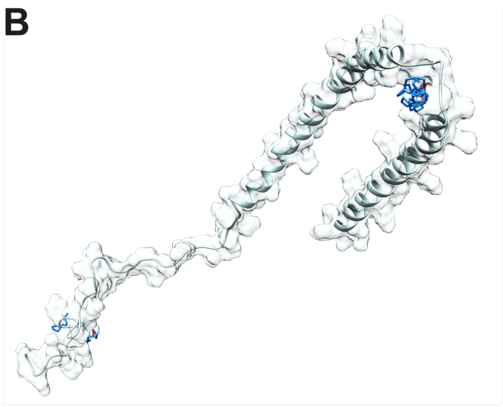

C

D

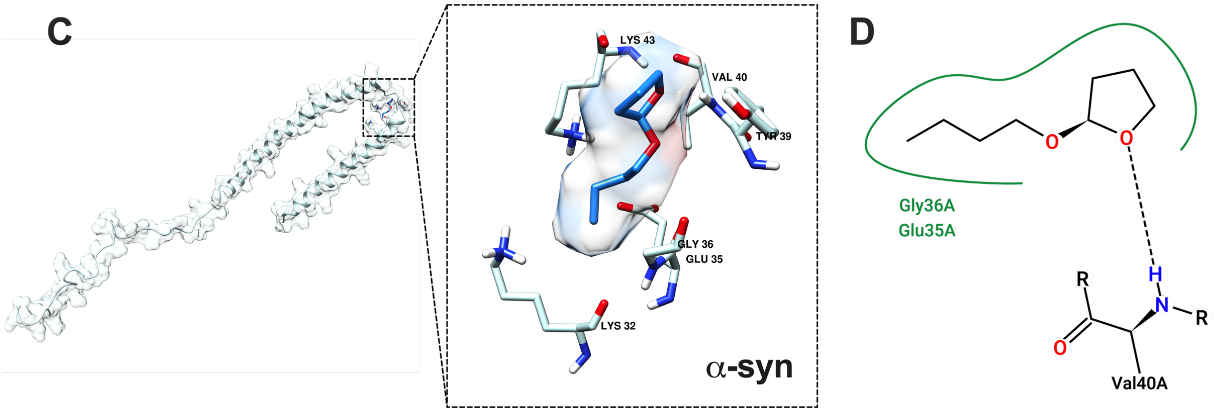

Figure S3. The interaction between 2-BTHF and α-synuclein was investigated through docking analysis. (A) Docking results displayed the top 10 scoring poses along with their respective RMSD values. (B) Image was generated to visualize all 10 conformations of the docked 2-BTHF onto the binding domain of α-synuclein. (C) Molecular coupling analysis unveiled the binding of the 2-BTHF molecule to active amino acid residues. (D) 2D structural depiction illustrated the interaction between 2-BTHF and α-synuclein, where hydrogen bonds were represented as dotted black line interactions, and hydrophobic contacts were indicated by green lines.

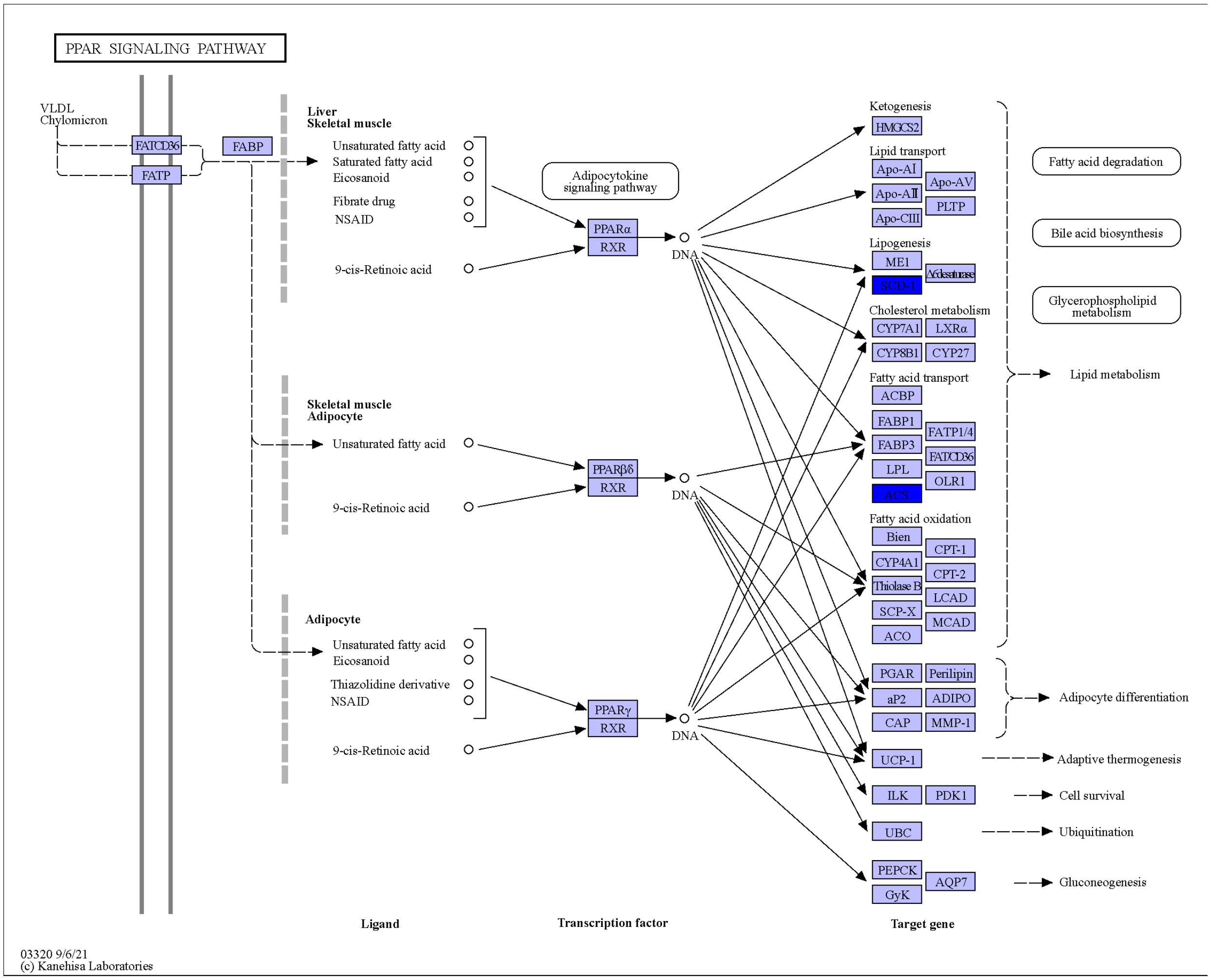

Figure S4. The exploration of differentially expressed genes (DEGs) was conducted through KEGG pathway analysis, focusing on the PPAR signaling pathway, which mediates ACS and SCD1 target genes associated with fatty acid metabolism.

Table S1. Docking information between 2-BTHF and human α-synuclein

Ligand	Protein	Interacting residues in		Docking energy score (kcal/mol)
		Hydrogen bond	Hydrophobic contact	
2-BTHF	α-synuclein	VAL 40	GLU 35, GLY 36	-3.5

Table S2. These are the primer sequences used for qRT-PCR analysis

C. elegans gene	Primer	Primer sequence (5' to 3')	NCBI sequence database
**HSF-1 associated gene**			
hsf-1	F	ATGCAGCCAGGATTGTCGAA	NM_060630.7
	R	GCACGTTTTGAGTTGGGTCC	
hsp-16.1	F	CGTCCAGCTCAACGTTCTGT	NM_072956.4
	R	TGGCTTGAACTGCGAGACAT	
hsp-16.2	F	GTCACTTTACCACTATTTCCGT	NM_001392482.1
	R	CAATCTCAGAAGACTCAGATGG	
hsp-16.49	F	CTCATGCTCCGTTCTCCATT	NM_072955.3
	R	GAGAAACATCGAGTTGAACAGAG	
hsp-70	F	AGCCCGTTGTTGAGGTTGAA	NM_060084.6
	R	CCCGTACAGAATGCCCAAGT	
ubc-9	F	GGAAACACTGGCGAAAGGATCA	NM_001027987.7
	R	TCGTCCTTGAAGAGCATCCG	
smo-1	F	AAGGTCGTTGGACAGGACAG	NM_058441.7
	R	CGTCATCGTCCTCCATCTCG	
ubh-4	F	GCACTTGTTCCAAACCGCAA	NM_001393132.1
	R	GACGTCGGCGATTGTTTTCC	
**Autophagy-lysosome gene**			
bec-1	F	AGATCTCAAAGCTGCGTGTG	NM_068443.4
	R	AAAAGGCAGAATTCCAGCAGA	
atg-7	F	TCTGCAGGATGGATGGTTCG	NM_069663.6
	R	CTCGGCAAGGTCCATGTGTA	
lgg-1	F	AATGGAAACCCAAAGCCCCT	NM_062876.8
	R	AGGGGAGAAGAGCAACTTCG	
lmp-1	F	CTCGCACCAACGAAGTTGTC	NM_076671.8
	R	GTATCCGACGAGCACAACCA	
**Fatty acid metabolism gene**			
acs-2	F	CCTCGAACATCCCAACCACA	NM_074468.6
	R	CGACACGATCTCCCTTCTCG	
fat-6	F	GCGCTGCTCACTATTTCGGATGG	NM_001268666.4
	R	GTGGGAATGTGTGATGGAAGTTGTG	
fat-7	F	CATGGAGGCAAACTCGACCT	NM_072413.7
	R	GTGGCGTGAAGTGTGAAACA	

**Glutathione system gene**			
gst-10	F	GTCTACCACGTTTTGGATGC	NM_071300.9
	R	ACTTTGTCGGCCTTTCTCTT	
gcs-1	F	AGGTGAATGCGATGCTTGGA	NM_063526.9
	R	CGATGAGACCTCCGTAAGGC	
gpx-4	F	GCCTGACTCATGTTCGTCCT	NM_067841.5
	R	AGCGGGTGTTGATTCTCTCC	
**Housekeeping gene**			
act-1	F	AGGTTGCCGCTCTTGTTGTA	NM_073418.9
	R	CGTGGTCTTCCGACAATGGA	

REFERENCES

1. Sanguanphun, T.; Promtang, S.; Sornkaew, N.; Niamnont, N.; Sobhon, P.; Meemon, K., Anti-Parkinson Effects of Holothuria leucospilota-Derived Palmitic Acid in Caenorhabditis elegans Model of Parkinson's Disease. *Marine Drugs* **2023,** *21* (3), 141.

2. Promtang, S.; Turbpaiboon, C.; Oo, E. M.; Khowawisetsut, L.; Uawithya, P.; Chompoopong, S., Germinated brown rice protects against glutamate toxicity in HT22 hippocampal neurons through the jnk-mediated apoptotic pathway via the GABAA receptor. *IBRO Neuroscience Reports* **2023,** *14*, 38-49.
